# GLP-1RAs caused gastrointestinal adverse reactions of drug withdrawal: a system review and network meta-analysis

**DOI:** 10.3389/fendo.2023.1149328

**Published:** 2023-07-06

**Authors:** Ziqi Zhang, Qiling Zhang, Ying Tan, Yu Chen, Xiqiao Zhou, Su Liu, Jiangyi Yu

**Affiliations:** ^1^ Department of Endocrinology, Jiangsu Provincial Hospital of Chinese Medicine, Affiliated Hospital of Nanjing University of Traditional Chinese Medicine, Nanjing, Jiangsu, China; ^2^ The First Clinical Medical College of Nanjing University of Chinese Medicine, Nanjing, China

**Keywords:** Glucagon-like peptide-1 receptor agonist, intolerance, gastrointestinal adverse effects, network meta-analysis, Dulaglutide, Liraglutide, Semaglutide

## Abstract

**Background:**

Glucagon-like peptide-1 receptor agonists (GLP-1RAs) significantly reduce postprandial blood glucose, inhibit appetite, and delay gastrointestinal emptying. However, it is controversial that some patients are intolerant to GLP-1RAs.

**Methods:**

PubMed, Embase, Web of Science, and Cochrane Library were searched for randomized controlled trials (RCTs) using GLP-1RAs with documented withdrawal due to gastrointestinal adverse reactions (GI AEs) from their inception to September 28, 2022. After extracting the information incorporated into the studies, a random-effects network meta-analysis was performed within a frequentist framework.

**Results:**

64 RCTs were finally enrolled, which included six major categories of the GLP-1RA. The sample size of the GLP-1RAs treatment group was 16,783 cases. The risk of intolerable gastrointestinal adverse reactions of Liraglutide and Semaglutide was higher than that of Dulaglutide. Meanwhile, the higher the dose of the same GLP-1RA preparation, the more likely to cause these adverse reactions. These intolerable GI AEs were not significantly related to drug homology or formulations and may be related to the degree of suppression of the appetite center.

**Conclusion:**

Dulaglutide caused the lowest intolerable GI AEs, while Liraglutide and Semaglutide were the highest. For Semaglutide, the higher the dose, the more likely it is to drive GI AEs. Meanwhile, the risk of these GI AEs is independent of the different formulations of the drug. All these findings can effectively guide individualized treatment.

**Systematic review registration:**

https://www.crd.york.ac.uk/prospero/display_record.php?ID=CRD42022359346, identifier CRD42022359346.

## Highlights

1. This Meta-Analysis selected people with intolerable GI AEs to withdraw from the Research.

2. GLP-1RAs are more likely to cause intolerable GI AEs than other hypoglycemic agents.

3. Among the six major categories of GLP-1RA, dulaglutide has the least risk of causing intolerable GI adverse reactions, and conversely, semaglutide has the highest chance of causing intolerable GI adverse reactions. Liraglutide has a similar risk of causing intolerable GI adverse reactions to Semaglutide.

4. The occurrences of intolerable GI AEs are related to the dose and type of drug and are not significantly related to drug homology or type of formulation but may be associated with the degree of suppression of the appetite center.

## Introduction

In 1993, exendin-4, which showed the same primary effect as endogenous GLP-1, was isolated from *Heloderma suspectum* venom (1). In 2005, Exenatide was approved for marketing by the U.S. Food and Drug Administration (FDA), indicating a new tool in the fight against diabetes. These individual GLP-1RAs have their strengths and shortcomings following their different physiochemical characteristics. Although the clinical efficacy of GLP1-RAs is unquestionable, the previous study (2) showed that the rate of HbA1c control in real-world patients deviated from the results of randomized controlled trials, which may be attributed to poor medication adherence. Many factors affect medication compliance, and adverse reactions are a common reason for poor patient compliance (3).

Juris et al. suggested that GLP-1RAs only mildly increased the risk of pancreatitis while this trend was not significant (4); however, no study has determined which GLP-1RAs are more tolerable due to these unknown intolerable adverse reactions. Monami et al. found that GLP-1RAs significantly increase the risk of gallstone disease (5). A meta-analysis of GI AEs, mainly based on the HARMONY series of trials, showed that the GI tolerability of albiglutide was lower than that of liraglutide, while the numbers of included literature were small, and the results were too single (6); Lin Xia et al. only compared the results of drop-out due to AEs of GLP-1RAs with placebo (7); Htike et al. showed higher risk for GI adverse effects with GLP-1RA versus placebo, with the lowest risk for nausea and diarrhea with Albiglutide and lowest risk for vomiting with the weekly Exenatide formulation (8). Still, these findings are of little clinical significance. Nausea, vomiting, or diarrhea could have led to discontinuation of the drug, so there is not necessary to compare these adverse reactions separately.

According to the current research findings, the mechanism of action of GLP-1RAs is similar, but due to their structural differences, the clinical-specific efficacy or adverse effects are biased. Researchers have found that the most effective GLP-1RA in lowering glucose and reducing weight is Semaglutide, the most effective GLP-1RAs in lowering postprandial glucose is Lixisenatide or Exenatide b.i.d, and the most convenient GLP-1RAs is Dulaglutide (9).

Meta-Analysis serves as the top level of evidence to guide clinical medication. This Network Meta-Analysis aimed to compare the risk of shedding different GLP-1RAs due to GI adverse reactions for providing evidence-based medical evidence for clinicians, policymakers, and guidelines deciding to choose one kind of GLP-1RAs.

## Methods

### Systematic literature review and outcome measures

This Network Meta-Analysis was guided and performed by the Preferred Reporting Items for Systematic Reviews and Meta-Analysis (PRISMA) guidelines, and a prospective protocol was developed and registered with PROSPERO (https://www.crd.york.ac.uk) under (ID: CRD42022359346). We searched PubMed, Embase, Web of Science, and the Cochrane Library for relevant English literature, limited to randomized clinical trials (RCTs). The following search keywords were as follows: (glucagon-like peptide-1 receptor agonist) or (GLP-1RA) or (Exenatide) or (Dulaglutide) or (Semaglutide) or (Liraglutide) or (Lixisenatide) or (Benaglutide) or (Albiglutide) or (Loxenatide). The retrieval time was from the establishment date of the database to September 28, 2022. The primary outcome indicator included intolerable GI AEs, including but not limited to nausea, vomiting, abdominal pain, diarrhea, pancreatitis, and cholelithiasis.

The inclusion criteria were as follows:

(1) RCTs
(2) at least one GLP-1RA,
(3) the primary outcome included intolerable gastrointestinal adverse effects and specified the number of cases dropped due to a specific gastrointestinal negative impact,
(4) In addition, studies in which GI AEs were tolerated without discontinuation;

The exclusion criteria were as follows:

(1) Animal studies; Self-control research; Dissertations and conference reports;
(2) Non-English literature;
(3) Other articles whose main text could not be retrieved;
(4) Retracted articles.

### Statistical methods and data synthesis

Two authors performed the literature selection, data extraction, and quality evaluation independently. Two authors screened each paper according to inclusion and exclusion criteria to exclude the possibility of errors(Zhang Ziqi, Zhang Qiling, Chen yu, Tan ying). If there is a dispute, the decision will be made by the third author with seniority(Liu su). Excel software was used to extract data, including the title, author, publication year, trial period, experimental and control groups’ intervention measures, the number of dropped cases, sample size, average age, average duration, Region, and other information. The Risk of bias table tool in Revman5.4.1(Review Manager (RevMan) [Computer program]. Version 5.4.1, The Cochrane Collaboration, 2020) was used to evaluate the methodological quality of each included study according to the Cochrane Collaboration Risk of bias tool (version 5.1.0). The methodological quality of each included study was assessed in terms of 7 aspects: generation of randomized sequences, allocation concealment, blinding of investigators and subjects, blinding of outcome indicators, completeness of outcome data, reporting bias, and other preferences. Literature quality evaluation of the included literature was performed using GRADE profiler 3.6.1. The Grading of Recommendations Assessment, Development, and Evaluation (GRADE) (10) was applied to assess the quality of each study in six primary areas: inter-study bias, reporting bias, indirectness, imprecision, heterogeneity, and inconsistency. All trials were considered to be of “low risk,” “some concern,” or “high risk.”

After data extraction and quality evaluation, network meta-analysis and mapping were performed using the network package and mvmeta package in Stata 16.0 and netmeta package in R 4.1.2 based on the frequentist framework (11, 12). Our study was divided into four main steps. STEP1, a Network Meta-Analysis was conducted to compare GLP-1RAs as a whole versus non-GLP-1RAs hypoglycemic agents. STEP2, GLP-1RAs specific drugs and non-GLP-1RAs hypoglycemic drugs were analyzed. In STEP2, we unified five preparations of Exenatide, Loxenatide (PEX168), ITCA650, Lixisenatide, and Efpeglenatide modified based on Exenatide-4 as Exenatide for analysis. STEP3, we selected Exenatide and Semaglutide, which exist in different dosage forms, for the network meta-analysis by dosage form classification. STEP4, a network meta-analysis of Semaglutide weekly preparations, was conducted based on dose to determine whether the incidence of intolerable gastrointestinal adverse reactions is related to the dose.

Stata 16.0 software was used to pre-process the data, plot the network relationship, perform Network Meta-Analysis, calculate relative effects, and draw a Ranking of risks. The publication bias was identified by drawing corrected comparison funnel plots. The inconsistency of the Network Meta-Analysis results was tested using the node-splitting method and the loop inconsistency test. *P*>0.05 for the difference between direct and indirect comparison results was considered insignificant. Thus, the consistency model was used. If the prediction interval crossed the null line, the random-effect model was applied due to the existing heterogeneity. If there was no significant heterogeneity, the fixed-effect model was used. The incidence of GI AEs was a dichotomous outcome, so the relative effect value of the result was expressed as OR and 95% CI. Risk ranking using surface under the cumulative ranking curve(SUCRA) (13) assessment, a greater SUCRA represents a higher risk of occurrence of the drug class in this outcome. *P*<0.05 was considered a statistically significant difference.

## Results

### Search results

**Figure 1:**
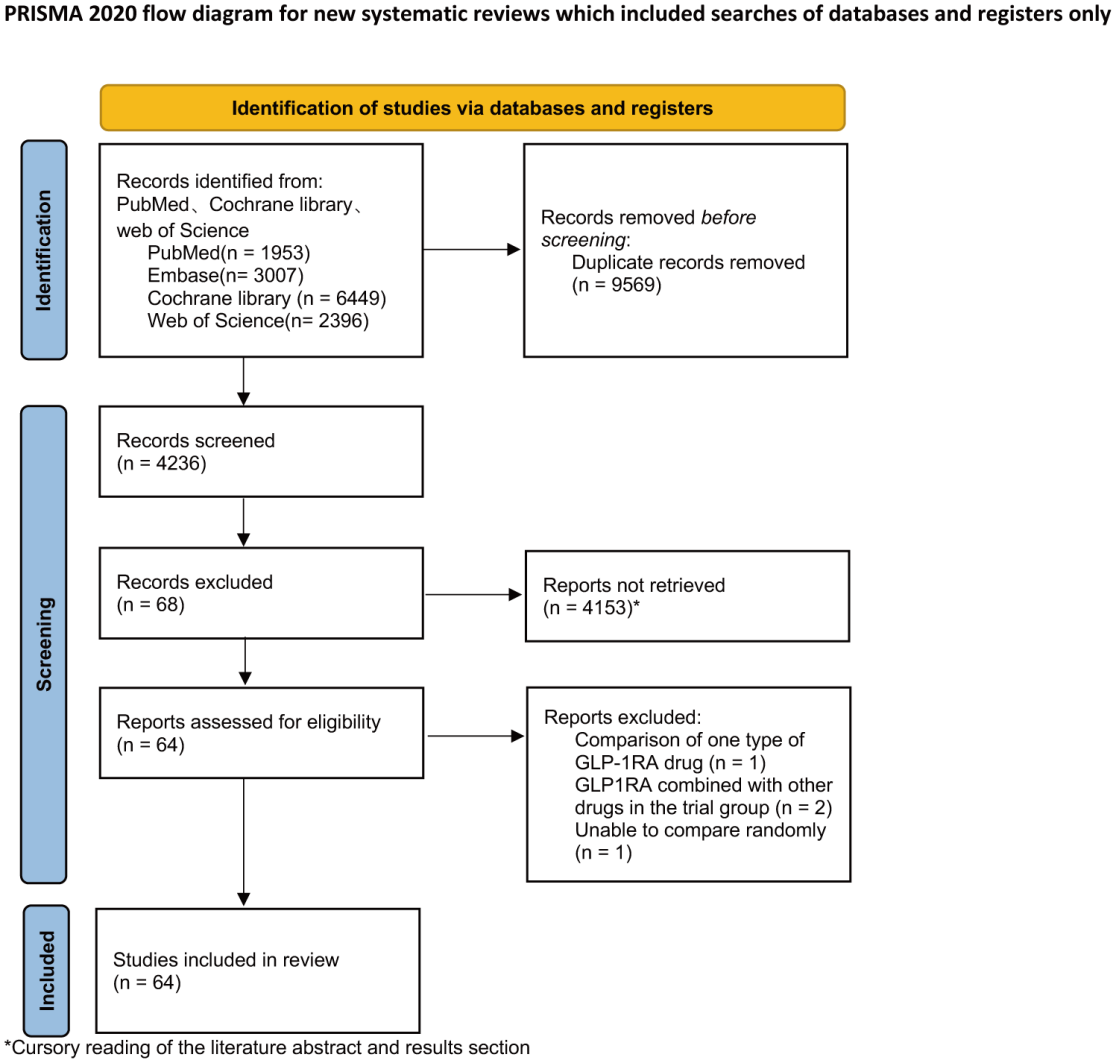
Flowchart of the literature search for this network meta-analysis.

A total of 13805 articles were searched in PubMed, Embase, Web of Science, and Cochrane Library, and 64 papers were finally included, all of which were RCTs. The screening procedure is shown in **Figure 1**.

### Eligible studies and patient characteristics

The essential characteristics and relevant information of the 64 included studies are detailed in **Table 1**. Of these, 55 were two-armed, 7 were three-armed, and 2 were four-armed. GLP-1RAs consisting of Exenatide, Liraglutide, Dulaglutide, Semaglutide, Tirzepatide, and Taspoglutide were included in the study. Among them, Exenatide includes six different dosage forms: Exenatide b.i.d, Exenatide q.w, PEX168, ITCA650, Lixisenatide, Efpeglenatide q.w, Efpeglenatide q.m. Semaglutide includes weekly formulation and oral formulation. Other hypoglycemic agents included metformin (Met), Insulin, sulfonylureas (SU), sodium-dependent glucose cotransporter 2 inhibitors (SGLT2i), dipeptidyl peptidase-IV inhibitor (DPP4i), thiazolidinedione (TZD). A total of 37740 patients were included in this study, and 16783 patients received GLP-1RAs. The shortest duration of the included studies was ten days, and the longest was 104 weeks. The mean age of the participants ranged from 14.5 to 74.2 years. Articles with missing data were excluded without contacting the authors for additional data.

**Table 1:** Characteristics of the included studies.

Author,year	Study population	Follow-up	Intervention	Total number of participants	Mean age(years)	Mean duration(years)	Region
Ahrén B (2017.05) (14)	T2DM	56week	Semaglutide 0.5 /1.0mg qw Sitagliptin 100mg qd	1225	55.1±10	6.6±5.1	Global multi-center
Aroda VR (2016.02) (15)	T2DM	30week	Semaglutide (0.5 mg/1.0 mg)qw Insulin glargine qd	1089	56.5±10.4	8.6±6.3	Global multi-center
Arslanian SA (2022.08) (16)	T2DM	26week	Dulaglutide 0.75mg/1.5mg qw Placebo	154	14.5±2.0	2.0±1.7	Global multi-center
Aso Y (2021.04) (17)	T2DM	52week	IDegLira qd Insulin Degludec and Insulin Aspart Injection bid	57	68.4±9.7	20.3±7.8	Japan multi-center
Astrup A (2009.11) (18)	OB	20week	Liraglutide 1.2/1.8/2.4/3.0mg qd Placebo Orlistat 120mg tid	564	45.9±10.3	NA	Europe multi-center
Barrington P (2011.05) (19)	T2DM	5week	Dulaglutide 0.05/0.3/1/3/5/8mg qd Placebo	43	55.3±6.1	NA	United States multi-center
Bergenstal RM (2010.08) (20)	T2DM	26week	Exenatide 2mg qw Sitagliptin 100 mg qd Pioglitazone 45mg qd	514	52.3±10.3	5.7±4.7	Global multi-center
Blonde L (2015.05) (21)	T2DM	52week	Dulaglutide 0.75mg/1.5mg qw Insulin glargine qd	884	59.4±9.2	12.7±7.0	Global multi-center
Brock C (2019.11) (22)	T1DM	26week	Liraglutide 1.2mg/1.8mg qd Placebo	48	50.4±8.6	32.4±9.3	Denmark single center
Buse JB (2013.01) (23)	T2DM	26week	Exenatide 2mg qw Liraglutide1.8mg qd	911	57±9.5	8.5±6	Global multi-center
Chen WR (2016.04) (24)	STEMI (after PCI)	once	Liraglutide 1.8mg Placebo	210	57.8±11.4	NA	China single center
D’Alessio D (2015.02) (25)	T2DM	24week	Liraglutide 1.8mg qd Insulin glargine qd	965	57±9	9±6	Global multi-center
Davies MJ (2009.12) (26)	T2DM	26week	Exenatide 10μg bid Insulin glargine 10u/d	235	56.5±9.1	8.7±4.5	UK multi-center
Davies M (2013.05) (27)	T2DM	26week	Exenatide 2mg qw Insulin detemir qd/bid	222	58.5±10	7.5±5.5	Europe multi-center
Derosa G (2013.08) (28)	T2DM	48week	Exenatide 10g bid Placebo	171	57.0±7.5	7.7±3.0	Italy multi-center
Frias JP (2019.09) (29)	T2DM	18week	Dulaglutide 1.5mg/3.0mg/4.5mg qw Placebo	318	56.8±9.7	8.0±6.2	Global multi-center
Frøssing S (2018) (30)	PCOS&OB/OW	26week	Liraglutide 1.8mg qd Placebo	72	29.9±6.1	NA	Copenhagen single center
Gao Y (2009.01) (31)	T2DM	16week	Exenatide 10mg bid Placebo	472	54±9	8±5	Asia multi-center
Grunberger (2012.10) (32)	T2DM	12week	Dulaglutide 0.1/0.5/1.0/1.5mgqw Placebo	167	56.6 ± 8.8	3.9 ± 3.7	Global multi-center
Gudipaty L (2014.09) (33)	T2DM	24week	Exenatide 10mg bid Sitagliptin 100mg qd Sitagliptin 100mg qd	47	55.3±2.7	3.9±1.0	United States single center
Hompesch M (2021.06) (34)	T2DM	12week	Exenatide 6mg qw/ 16mg qm Liraglutide 1.8mg qd	47	52.5±8.5	NA	United States single center
Husain M (2019.08) (35)	T2DM	36week	Oral Semaglutide 14mg qd Placebo	3183	66±7	14.9±8.5	Global multi-center
Inagaki N (2012.09) (36)	T2DM	26weeek	Exenatide 2mg qw Insulin glargine	427	56.8±10.8	9.03±6.02	Japan multi-center
J.Sever M (2014.03) (37)	PCOS&OB	12week	Liraglutide 1.2mg qd MET bid + LIRA 1.2 mg qd MET bid	36	39.3±4.2	NA	Slovenia single center
Jiang J (2011.12) (38)	HV	21day	Liraglutide 0.6/1.2/1.8mg qd Placebo	37	30 ± 6	NA	China single center
Jones KL (2020.05) (39)	HV	8week	Exenatide 2mg qw Placebo	35	60.3±0.7	NA	Australia multi-center
Koska J (2015.07) (40)	T2DM	11day	Exenatide 5-10ug bid Placebo	42	63±6	5.5	single center
Kothare PA (2008.12) (41)	T2DM	10day	Exenatide 2.5ug/5ug bid Placebo	40	53.0±8.9	NA	Japan single center
Kuhadiya ND (2016.07) (42)	T1DM	12week	Liraglutide 0.6/1.2/1.8 mg qd Placebo	72	NA	NA	New York, United States Single center
Li CJ (2014.09) (43)	T2DM	24week	Liraglutide 1.2mg qd Saxagliptin 5 mg qd Vildagliptin 50mg bid	203	NA	NA	China single center
Liu X (2017.12) (44)	PCOS& OB	12week	Exenatide 10ug bid Met 1000 mg bid	178	27.8±3.3	NA	China single center
Liutkus J (2010.12) (45)	T2DM	26week	Exenatide 10ug bid Placebo	165	54.7±8.3	6.3±4.3	Global multi-center
Ma RL (2021.11) (46)	PCOS&OB	12week	Exenatide 2mg qw+ Met tid Met tid	50	29.1±4.5	NA	China single center
Marso SP (2016.11) (47)	T2DM	104week	Semaglutide 0.5mg/1.0mg qw Placebo	3297	64.6±7.4	13.9±8.1	Global multi-center
Mathieu C (2014.07) (48)	T2DM	52week	Insulin degludec qd+ Liraglutide qd Insulin degludec qd+Insulin aspart qd	177	61±9.2	12.4±6.5	Global multi-center
Matikainen N (2019.01) (49)	T2DM&OB	16week	Liraglutide 1.8mg qd Placebo	22	62.3±2	7.2±5.3	Finland single center
Meneilly GS (2017.04) (50)	T2DM	24week	Lixisenatide 20 ug qd Placebo	350	74.2 ±3.9	14.1 ±7.6	Global multi-center
Miya A (2018.01) (51)	T2DM	12week	Lixisenatide 20ug qd +Basal insulin qd MDI(multiple daily insulin injection)	31	62.3 ±11.4	20.2±11.3	Japan multi-center
Miyagawa J (2015.01) (52)	T2DM	26week	Dulaglutide 0.75mg qw Liraglutide 0.9mg qd Placebo	487	57.4±9.6	6.6±5.6	Japan multi-center
Pfeffer MA (2015.12) (53)	T2DM&ACS	100week	Lixisenatide 20μg qd Placebo	6068	60.3±9.7	9.3±8.3	Global multi-center
Pi-Sunyer X (2015.07) (54)	OB	56week	Liraglutide 3.0mg qd Placebo	3731	45.1±12.0	NA	Global multi-center
Pozzilli P (2017.07) (55)	T2DM	28week	Dulaglutide 1.5 mg qw Placebo	300	60.4±9.8	13.2±7.6	Global multi-center
Riddle MC (2013.09) (56)	T2DM	24week	Lixisenatide 20ug qd Placebo	495	57±10	12.5±6.8	Global multi-center
Riddle MC(2013.09) (57)	T2DM	24week	lixisenatide 20ug qd Placebo	446	56±10	9.2±5.9	Global multi-center
Rodbard HW (2019.12) (58)	T2DM	52week	Semaglutide 14mg qd Empagliflozin 25 mg qd	822	58±10	7.4±6.1	Global multi-center
Rosenstock J (2013.03) (59)	T2DM	24week	Taspoglutide 10/20mg qw Exenatide 10mg bid	1149	55.7±9.8	6.6±5.4	Europe multi-center
Rosenstock J (2014.07) (60)	T2DM	24week	lixisenatide 20ug Placebo	859	57.3±9.9	9.3±6.0	Global multi-center
Rosenstock J (2016.09) (61)	T2DM	24week	LixiLan qd Insulin glargine qd	323	56.8±9.5	6.7±4.8	Global multi-center
Rosenstock J (2018.02) (62)	T2DM	39week	ITCA 650 40/60 mg qd Placebo	441	55.0±9.7	8.9±6.4	United States multi-center
Rosenstock J (2019.09) (63)	T2DM	12week	Efpeglenatide 0.3mg/1mg/2mg/3mg/4mg qw Liraglutide 1.8mg qd	254	55.1±10.0	6.1±5.1	Global multi-center
Rosenstock J (2021.07) (64)	T2DM	40week	Tirzepatide 5mg/10mg/15mg qw Placebo	478	54.1±11.9	4.7±5.4	Global multi-center
R.Jones D (2012.02) (65)	T2DM	26week	Exenatide 2mg qw Met 2000mg qd Pioglitazone 45mg qd Sitagliptin 100mg qd	820	53.8±11	2.7±3.5(?)	Global multi-center
Seino Y (2008.08) (66)	T2DM	14week	Liraglutide 0.1/0.3/0.6/0.9mg qd Placebo	226	57.3±8.1	7.6±5.4	Japan multi-center
Seino Y (2012.01) (67)	T2DM	24week	Lixisenatide 20μg qw Placebo	311	58.3±10.1	13.9±7.7	Asia multi-center
Shi XL (2017.05) (68)	T2DM	12weeek	STII+exenatide 10ug bid STII	129	45±8	NA	China multi-center
Sorli C (2017.04) (69)	T2DM	30week	Semaglutide 0.5 mg/1.0 mg qw Placebo	388	53.7±11.3	4.18±5.52	Global multi-center
Umpierrez G (2014.08) (70)	T2DM	52week	Dulaglutide 0.75mg/1.5mg qw Met qd	807	55.7±10.3	3±2	Global multi-center
V.Ruiten CC (2022.05) (71)	T2DM&OB	16week	Exenatide 10μg bid + Dapagliflozin 10mg Exenatide 10μg bid+placebo for Dapagliflozin Placebo for Exenatide+Dapagliflozin ± Met ± SU Placebo ± Met ± SU	65	63.5±0.9	8.4	Netherlands single center
Weinstock RS (2015.09) (72)	T2DM	104week	Dulaglutide 1.5/0.75 mg qw Sitagliptin100 mg Placebo	1098	54	7	Global multi-center
Xu W (2015.01) (73)	T2DM	48week	Exenatide 10ug bid Insulin qd	416	NA	0	China multi-center
Yamada Y (2017.09) (74)	T2DM	4week	Lixisenatide 20μg qd Sitagliptin 50mg qd	136	58.4±9.9	10.6	Japan multi-center
Yang GR (2015.02) (75)	T2DM	8week	Loxenatide 50ug/100ug/200ug/300ug qw Placebo	50	52.3±7.7	NA	China multi-center
Yang WY (2018.02) (76)	T2DM	24week	Lixisenatide 20μg qd Placebo	448	55.0±9.6	10.3 ±6.1	Asia multi-center
Zinman B (2007.04) (77)	T2DM	16week	Exenatide 10ug bid Placebo	233	56.1±10.5	7.7±5.3	Global multi-center

NA, Not Available.

### Quality assessment

**Figure 2:**
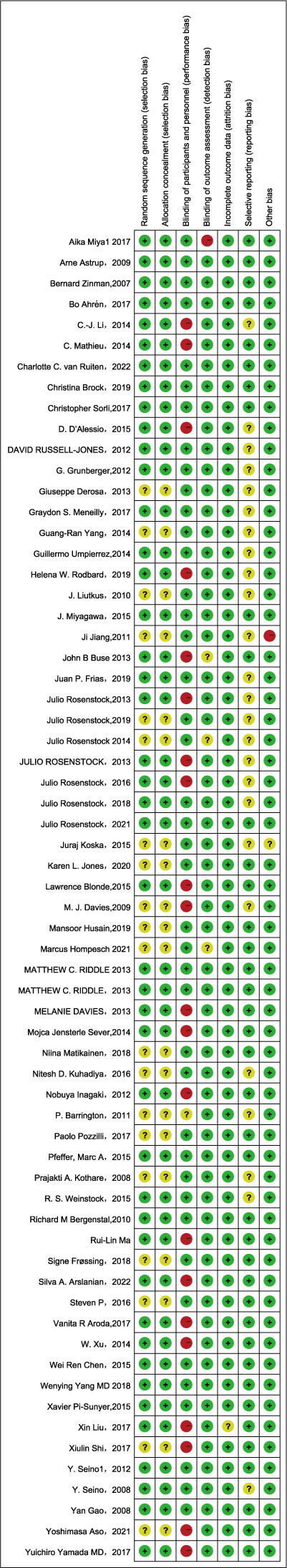
Risk of bias summary for each item and the included studies. (+) green circle, favorable; yellow circle, moderate (-).

All 64 included documents were RCTs, of which 21 studies did not specify the randomization method, and 1 study had both randomized and non-randomized parts. Based on inclusion and exclusion criteria, this study was classified as low risk; we ranked the 20 open-label papers as high risk because they have a problem with blinding participants and personnel. Blinding of outcome assessment, incomplete outcome data, and selective reporting were all satisfactory in all studies. After a comprehensive analysis, we concluded that the risk of bias in this included literature was low. According to the Cochrane Risk of Bias Assessment Tool, the result of discrimination was shown in **Figure 2**. GRADE ratings of the included literature showed that the vast majority of comparisons were low risk. This study was considered moderate quality evidence overall. The detailed results were established in the **Supplementary Materials**.

### Outcomes of STEP 1-4

**Figure 3:**
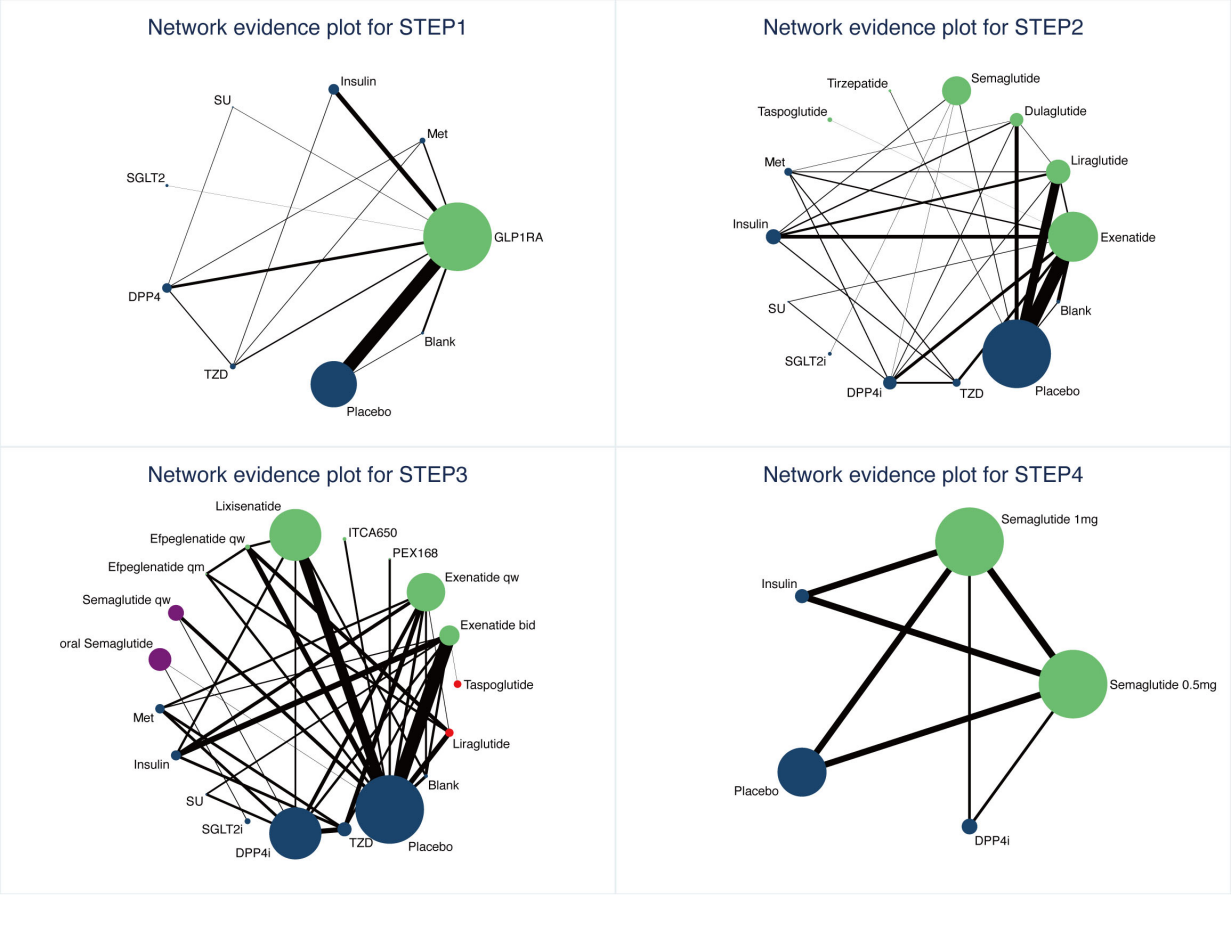
Network evidence plots for intolerable GI AEs.

**Figure 4:**
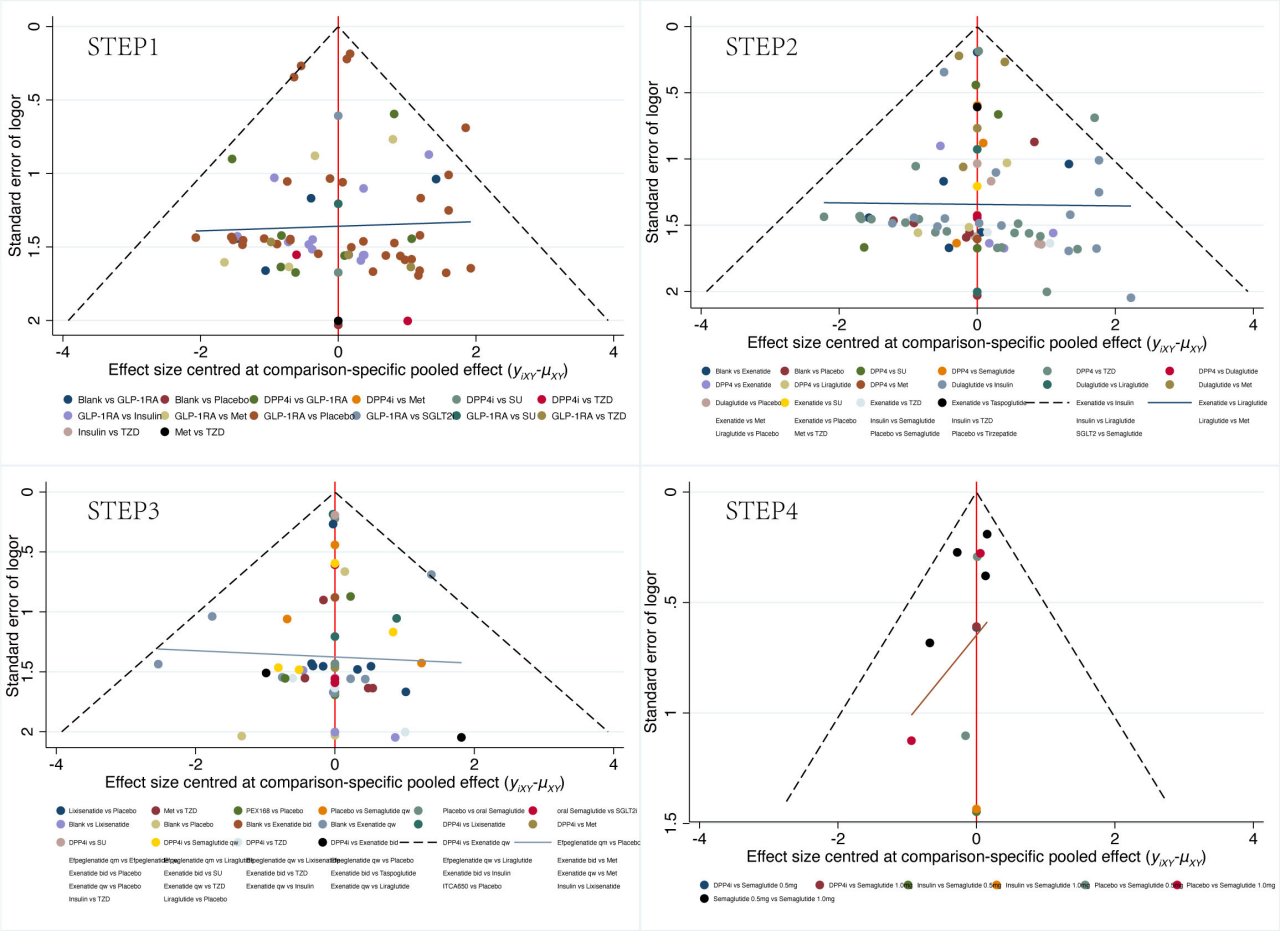
Comparison-adjusted funnel plots for STEP1-4.

**Figure 5:**
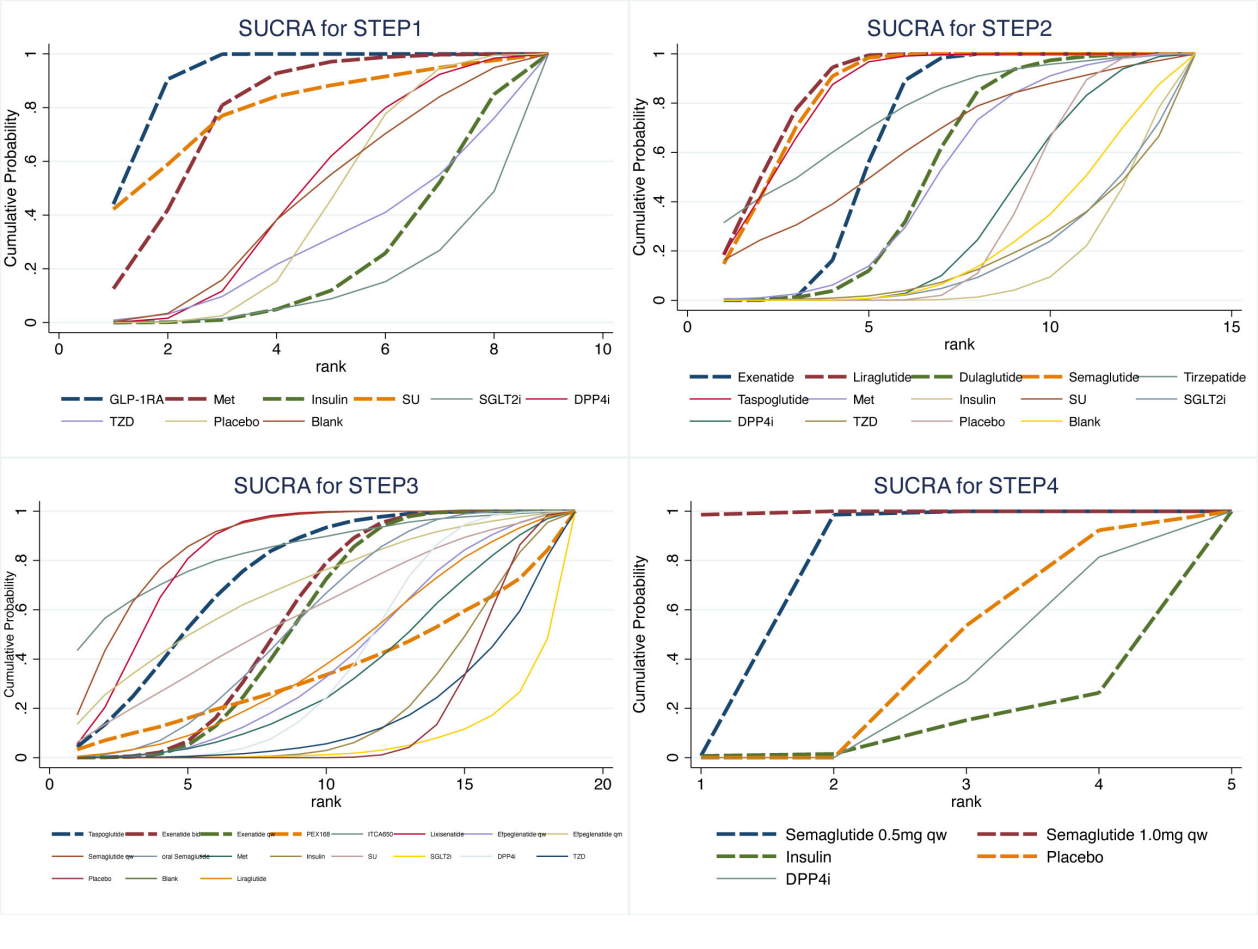
SUCRA for STEP1-4.

**Figure 6:**
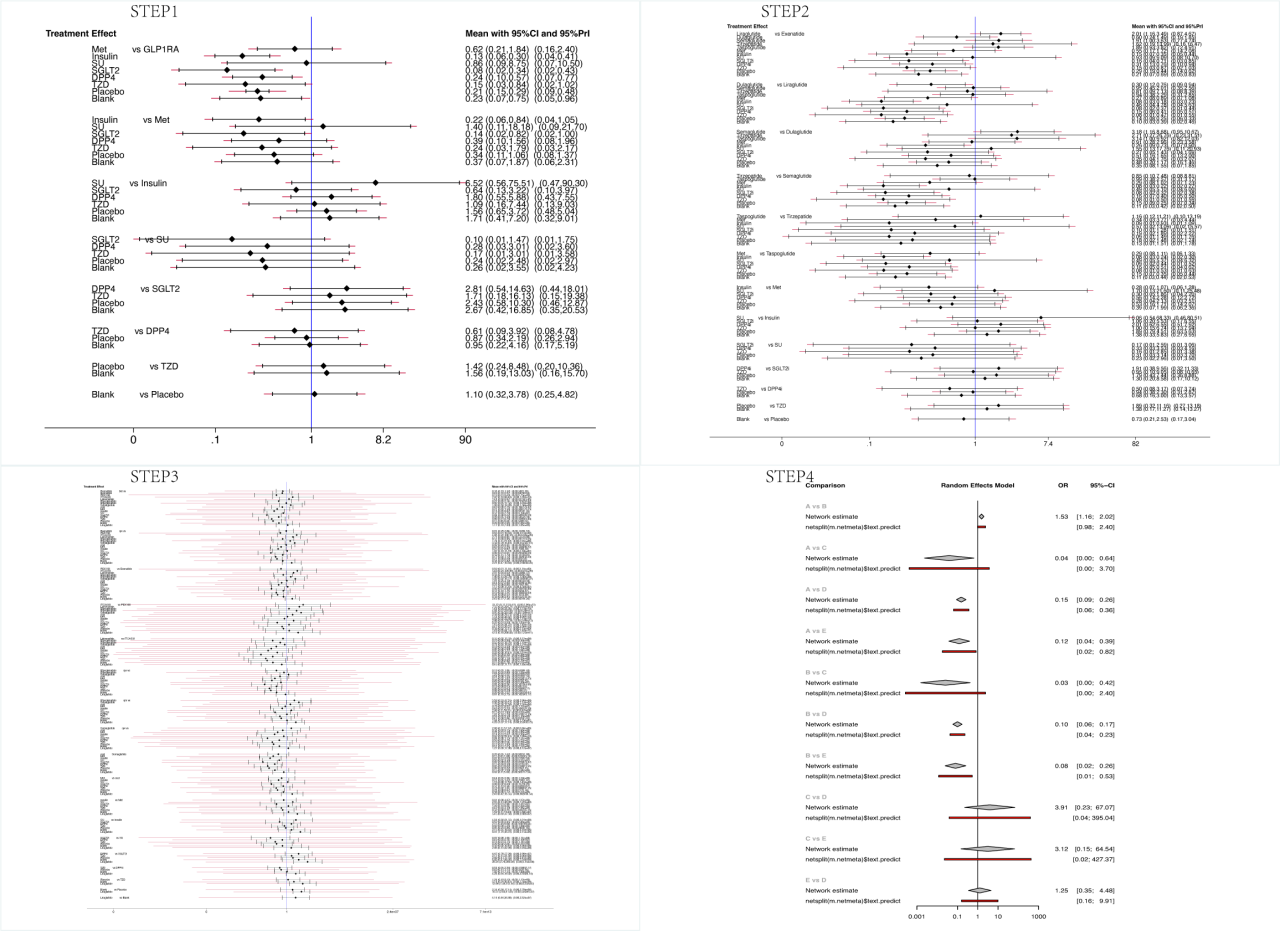
The interval plot for STEP1-4 (In STEP 4, A: Semaglutide 0.5mg qw; B: Semaglutide 1.0mg qw; C: Insulin; D: Placebo; E: DPP4i.).

In the reticulated body of evidence for STEP 1-4 (**Figure 3**) and the GLP-1RAs, eight control interventions, including Met, Insulin, SU, SGLT2i, DPP4i, TZD, Placebo, and Blank, were involved. The most significant direct comparison study with the GLP-1RAs was Placebo. The drug with the largest sample size among GLP-1RAs was Semaglutide. In this study, the four Network maps of comparisons included in the analyses were tested using the loop inconsistency test and the node-splitting method. No significant inconsistency or heterogeneity was found. We performed the Network Meta-Analysis in a random-effects model with a consistency model. The results of each Network league table in STEP1-4 are shown in **Table 2**. A funnel plot assessing the risk of publication bias showed symmetric distribution, indicating a low risk of publication bias(**Figure 4**). SUCRAs for all results are available in **Figure 5**. Prediction interval plots also showed low heterogeneity among studies (**Figure 6**).

**Table 2:** Network league table.

GLP-1RA																		
1.63 (0.54,4.87)	Met			**STEP1**														
7.54 (3.39,16.78)	4.64 (1.20,17.99)	Insulin																
1.16 (0.11,11.72)	0.71 (0.06,9.21)	0.15 (0.01,1.78)	SU															
11.78 (2.90,47.90)	7.25 (1.22,43.02)	1.56 (0.31,7.85)	10.19 (0.68,152.60)	SGLT2														
4.20 (1.76,10.05)	2.58 (0.64,10.40)	0.56 (0.17,1.82)	3.63 (0.33,39.61)	0.36 (0.07,1.86)	DPP4													
6.89 (1.19,39.80)	4.24 (0.56,32.28)	0.91 (0.13,6.22)	5.96 (0.33,106.95)	0.59 (0.06,5.52)	1.64 (0.25,10.58)	TZD												
4.84 (3.45,6.79)	2.98 (0.94,9.45)	0.64 (0.27,1.54)	4.19 (0.40,43.42)	0.41 (0.10,1.74)	1.15 (0.46,2.91)	0.70 (0.12,4.18)	Placebo											
4.41 (1.34,14.58)	2.72 (0.53,13.79)	0.59 (0.14,2.47)	3.82 (0.28,51.64)	0.37 (0.06,2.36)	1.05 (0.24,4.60)	0.64 (0.08,5.34)	0.91 (0.26,3.14)	Blank										
Exenatide																		
0.50 (0.29,0.86)	Liraglutide			**STEP2**														
1.67 (0.67,4.13)	3.36 (1.31,8.57)	Dulaglutide																
0.52 (0.28,1.00)	1.06 (0.50,2.24)	0.31 (0.12,0.86)	Semaglutide															
0.62 (0.07,5.31)	1.24 (0.14,10.98)	0.37 (0.04,3.66)	1.18 (0.13,10.34)	Tirzepatide														
0.53 (0.26,1.07)	1.07 (0.44,2.62)	0.32 (0.10,1.00)	1.01 (0.39,2.63)	0.86 (0.09,8.31)	Taspoglutide													
1.83 (0.58,5.74)	3.68 (1.12,12.12)	1.10 (0.34,3.56)	3.49 (0.98,12.43)	2.97 (0.27,33.24)	3.45 (0.90,13.22)	Met												
6.52 (2.83,15.03)	13.14 (5.47,31.56)	3.91 (1.36,11.24)	12.45 (4.65,33.35)	10.59 (1.08,104.03)	12.30 (4.12,36.69)	3.57 (0.93,13.61)	Insulin											
1.08 (0.11,10.55)	2.17 (0.21,22.52)	0.65 (0.06,7.42)	2.05 (0.20,21.52)	1.75 (0.08,40.00)	2.03 (0.19,22.12)	0.59 (0.05,7.49)	0.17 (0.01,1.86)	SU										
6.18 (1.41,27.09)	12.44 (2.70,57.43)	3.71 (0.70,19.64)	11.78 (3.11,44.59)	10.03 (0.78,128.27)	11.64 (2.26,59.91)	3.38 (0.54,21.26)	0.95 (0.18,4.96)	5.73 (0.39,85.30)	SGLT2i									
3.24 (1.32,7.96)	6.53 (2.42,17.63)	1.94 (0.61,6.24)	6.18 (2.48,15.38)	5.26 (0.53,52.25)	6.11 (1.95,19.15)	1.77 (0.44,7.15)	0.50 (0.15,1.62)	3.01 (0.28,31.95)	0.52 (0.10,2.63)	DPP4i								
6.53 (1.15,37.22)	13.16 (2.15,80.71)	3.92 (0.57,27.02)	12.47 (2.01,77.34)	10.60 (0.67,167.43)	12.32 (1.88,80.52)	3.57 (0.47,27.22)	1.00 (0.15,6.76)	6.07 (0.35,104.95)	1.06 (0.11,10.13)	2.02 (0.32,12.88)	TZD							
3.45 (2.27,5.24)	6.95 (4.07,11.86)	2.07 (0.85,5.03)	6.58 (3.95,10.96)	5.60 (0.68,46.35)	6.50 (2.87,14.76)	1.89 (0.59,6.08)	0.53 (0.22,1.26)	3.20 (0.32,32.25)	0.56 (0.13,2.32)	1.06 (0.43,2.61)	0.53 (0.09,3.11)	Placebo						
4.73 (1.46,15.37)	9.54 (2.60,35.03)	2.84 (0.64,12.52)	9.03 (2.38,34.27)	7.68 (0.66,89.20)	8.92 (2.26,35.22)	2.59 (0.50,13.36)	0.73 (0.17,3.07)	4.40 (0.34,57.24)	0.77 (0.12,5.04)	1.46 (0.33,6.40)	0.72 (0.09,5.92)	1.37 (0.40,4.75)	Blank					
Taspoglutide																		
1.88 (0.66,5.38)	Exenatide b.i.d			**STEP3**														
2.06 (0.44,9.69)	1.10 (0.35,3.41)	Exenatide q.w																
5.24 (0.15,177.48)	2.78 (0.10,80.28)	2.54 (0.09,74.40)	PEX168															
0.38 (0.01,10.00)	0.20 (0.01,4.46)	0.19 (0.01,4.14)	0.07 (0.00,5.98)	ITCA650														
0.69 (0.14,3.36)	0.37 (0.11,1.20)	0.34 (0.11,1.07)	0.13 (0.00,3.94)	1.82 (0.08,41.34)	Lixisenatide													
3.67 (0.47,28.80)	1.95 (0.33,11.48)	1.78 (0.37,8.45)	0.70 (0.02,26.39)	9.60 (0.33,282.06)	5.28 (0.96,29.06)	Efpeglenatide q.w												
1.09 (0.06,20.63)	0.58 (0.04,9.03)	0.53 (0.04,7.40)	0.21 (0.00,13.67)	2.84 (0.05,151.34)	1.56 (0.10,24.33)	0.30 (0.02,4.15)	Efpeglenatide q.m											
0.53 (0.08,3.35)	0.28 (0.06,1.29)	0.26 (0.06,1.09)	0.10 (0.00,3.38)	1.38 (0.05,35.82)	0.76 (0.19,3.05)	0.14 (0.02,1.04)	0.49 (0.03,8.84)	Semaglutide q.w										
2.05 (0.37,11.46)	1.09 (0.28,4.26)	0.99 (0.24,4.03)	0.39 (0.01,12.01)	5.36 (0.23,126.26)	2.95 (0.70,12.44)	0.56 (0.08,3.86)	1.89 (0.11,32.56)	3.89 (0.70,21.47)	oral Semaglutide									
4.66 (0.63,34.55)	2.47 (0.45,13.63)	2.26 (0.34,14.90)	0.89 (0.02,37.03)	12.19 (0.37,398.55)	6.71 (0.94,48.10)	1.27 (0.12,13.35)	4.29 (0.18,101.36)	8.85 (1.00,78.42)	2.28 (0.27,18.85)	Met								
7.61 (1.49,38.97)	4.04 (1.16,14.13)	3.69 (1.07,12.69)	1.45 (0.04,47.84)	19.92 (0.78,505.96)	10.97 (2.63,45.68)	2.08 (0.31,13.89)	7.01 (0.41,121.34)	14.45 (2.56,81.71)	3.72 (0.70,19.65)	1.63 (0.21,12.47)	Insulin							
1.76 (0.13,24.10)	0.93 (0.08,10.27)	0.85 (0.07,11.13)	0.34 (0.01,20.08)	4.60 (0.10,221.11)	2.53 (0.20,32.64)	0.48 (0.03,8.77)	1.62 (0.04,58.95)	3.34 (0.22,51.08)	0.86 (0.06,12.81)	0.38 (0.02,6.99)	0.23 (0.02,3.29)	SU						
24.12 (2.39,243.26)	12.80 (1.63,100.37)	11.68 (1.45,93.90)	4.60 (0.11,196.91)	63.12 (1.88,2123.40)	34.75 (4.22,286.29)	6.58 (0.56,77.97)	22.22 (0.87,566.75)	45.80 (4.59,457.33)	11.78 (2.52,55.03)	5.18 (0.38,70.84)	3.17 (0.33,30.62)	13.71 (0.61,307.49)	SGLT2i					
3.62 (0.72,18.24)	1.92 (0.56,6.58)	1.75 (0.58,5.30)	0.69 (0.02,21.07)	9.46 (0.40,221.42)	5.21 (2.21,12.30)	0.99 (0.17,5.61)	3.33 (0.21,52.55)	6.87 (1.98,23.75)	1.77 (0.39,7.91)	0.78 (0.11,5.57)	0.47 (0.11,2.03)	2.06 (0.16,25.93)	0.15 (0.02,1.29)	DPP4i				
11.10 (1.28,95.91)	5.89 (0.89,38.77)	5.37 (0.82,35.06)	2.12 (0.05,90.87)	29.04 (0.86,980.07)	15.98 (2.22,115.06)	3.03 (0.29,32.05)	10.22 (0.43,244.70)	21.07 (2.37,187.09)	5.42 (0.62,47.29)	2.38 (0.22,26.04)	1.46 (0.18,11.61)	6.31 (0.32,125.34)	0.46 (0.03,6.57)	3.07 (0.44,21.29)	TZD			
8.98 (2.33,34.60)	4.76 (2.04,11.13)	4.35 (1.76,10.77)	1.71 (0.07,44.35)	23.50 (1.20,459.36)	12.93 (4.94,33.86)	2.45 (0.49,12.25)	8.27 (0.59,115.89)	17.05 (4.50,64.57)	4.39 (1.50,12.80)	1.93 (0.31,11.93)	1.18 (0.33,4.22)	5.10 (0.43,60.97)	0.37 (0.06,2.43)	2.48 (0.87,7.09)	0.81 (0.12,5.32)	Placebo		
3.53 (0.38,32.87)	1.88 (0.26,13.42)	1.71 (0.25,11.59)	0.67 (0.02,29.33)	9.25 (0.27,316.66)	5.09 (0.73,35.40)	0.96 (0.09,10.34)	3.26 (0.13,79.02)	6.71 (0.73,62.10)	1.73 (0.19,15.42)	0.76 (0.06,9.49)	0.46 (0.05,3.95)	2.01 (0.10,41.96)	0.15 (0.01,2.13)	0.98 (0.13,7.22)	0.32 (0.02,4.10)	0.39 (0.06,2.66)	Blank	
0.85 (0.13,5.55)	0.45 (0.10,2.13)	0.41 (0.13,1.29)	0.16 (0.00,5.55)	2.24 (0.08,58.85)	1.23 (0.27,5.65)	0.23 (0.06,0.93)	0.79 (0.06,10.54)	1.62 (0.28,9.52)	0.42 (0.07,2.36)	0.18 (0.02,1.60)	0.11 (0.02,0.58)	0.49 (0.03,7.78)	0.04 (0.00,0.36)	0.24 (0.05,1.06)	0.08 (0.01,0.67)	0.10 (0.02,0.37)	0.24 (0.03,2.16)	Liraglutide
Semaglutide 0.5mg q.w																		
0.66 (0.47,0.91)	Semaglutide 1.0mg q.w			**STEP4**														
25.68 (1.55,425.32)	39.07 (2.37,644.60)	Insulin																
6.55 (3.77,11.37)	9.96 (5.71,17.36)	0.25 (0.01,4.41)	Placebo															
8.25 (2.51,27.05)	12.55 (3.84,40.98)	0.32 (0.02,6.69)	1.26 (0.35,4.57)	DPP4i														

Positive results were highlighted in blue.

#### STEP 1

Sixty-one studies were included in STEP 1. The network evidence map is shown in **Figure 3**. STEP 1 results showed that GLP-1RA preparations differed from other hypoglycemic agents or placebo, except for Met and SU. GI AEs analysis found that GLP-1RAs showed a higher risk compared to Insulin (OR=7.54, 95%CI 3.39, 16.78), SGLT2i(OR=11.78, 95%CI 2.90, 47.90), DPP4i(OR=4.20, 95%CI 1.76, 10.05), TZD(OR=6.89, 95%CI 1.19, 39.80), Placebo(OR=4.84, 95%CI 3.45, 6.79) and Blank(OR=4.41, 95%CI 1.34, 14.58). Also, we observed that Met caused a higher risk of GI AEs than Insulin (OR=4.64. 95%CI 1.20, 17.99) and SGLT2i (OR=7.25, 95%CI 1.22, 43.02). Risk ranking and SUCRA analysis showed that GLP-1RAs ranked first (91.8), followed by SU (79.3), Met (78.0), DPP4i (48), Blank (45.3), Placebo (41.9), TZD (29.8), Insulin (22.6), and SGLT2i(13.4) had the lowest rate of intolerable GI AEs. The high incidence of adverse reactions with SU may be related to the fact that only one study intervention included SU. Therefore, we do not consider this result to be clinically meaningful. Overall, the results of STEP1 showed that GLP-1RAs were more prone to higher intolerable GI AEs than Insulin, DPP4i, TZD, Placebo, and especially SGLT2i.

#### STEP 2

Sixty-four studies were included in STEP 2. The network evidence map was shown in **Figure 3**. The results of STEP 2 indicate that Exenatide has a lower risk of causing GI AEs than Liraglutide and Semaglutide (OR=0.50 95%CI 0.29,0.86), (OR=0.52 95%CI 0.28,1.00); Liraglutide has a higher risk of causing intolerable GI adverse reactions compared to Dulaglutide (OR=3.36 95%CI 1.31,8.57); Dulaglutide has a lower risk of GI AEs compared to Semaglutide, Taspoglutide (OR=0.31 95%CI 0.12,0.86), (OR=0.32 95%CI 0.10,1.00). Meanwhile, the odds ratios and confidence intervals of Exenatide, Liraglutide, Semaglutide, and Taspoglutide all had statistically significant risks of GI AEs compared to placebo (OR=3.45 95%CI 2.27,5.24), (OR=6.95 95%CI 4.07,11.86), (OR=6.58 95%CI 3.95,10.96), and (OR=6.50 95%CI 2.87,14.76). Risk ranking showed that Liraglutide (87.7) ranked first, followed by Semaglutide (85.8), Taspoglutide (85.4), Tirzepatide (76.4), Exenatide (66.3), Dulaglutide (52.7), Placebo (30.9). The results of STEP2 showed that Dulaglutide had the lowest risk of intolerable GI AEs. Significantly, Liraglutide and Semaglutide have similar SUCRAs.

#### STEP 3

Forty studies were included in STEP 3. The network evidence diagram is shown in **Figure 3**. STEP 3 results showed no significant difference between various preparations of Exenatide and Semaglutide. The risk ranking showed that Semaglutide q.w (87.1) ranked first, followed by ITCA650 (83.8), Lixisenatide (83.2), Liraglutide (78.1), Taspoglutide (74.1), Efpeglenatide q.m (68.4), Exenatide b.i.d (57.3), oral Semaglutide (55.3), Exenatide q.w (54.9), Efpeglenatide q.w (39.4), and PEX168 (35.8), Placebo (16.5), while PEX168(Loxenatide) had the lowest rate of GI adverse reactions. Although there was a sequential ranking between the different dosage forms of Exenatide and Semaglutide, we could not confirm the difference between the injectable and oral dosage forms of Semaglutide or the modified dosage form or method of use of Exenatide. The STEP3 results showed no significant difference in the incidence of intolerable GI AEs between disparate dosage forms of the same GLP-1RA and disparate modifications. In addition, we found that the incidence of intolerable GI AEs remained at the top for Semaglutide versus Liraglutide, thus confirming the results in STEP 2.

#### STEP 4

Four articles were included in STEP 4. **Figure 3** showed the network evidence plot result. STEP 4 results showed that Semaglutide 0.5 mg q.w caused a lower risk of intolerable GI AEs than Semaglutide (OR=0.66, 95%CI 0.47,0.91). The risk ranking results also showed that Semaglutide 1mg q.w (99.6) was associated with a higher risk than 0.5mg q.w (74.8). Meanwhile, the STEP 4 results showed that intolerable GI AEs were more pronounced with higher doses.

## Discussion

Research on GLP-1RAs over the past 30 years has been sufficient to confirm their place in glucose-lowering and weight loss (78, 79). Given the broad range of indications for GLP-1RAs, we included both diabetic and obese patients. Due to the remarkable efficacy of GLP-1RAs, it has been widely used in the clinic, and many adverse effects have occurred. GLP-1 receptors are expressed in several organs throughout the body, such as the intestine, heart, brain, kidneys, and even the peripheral nervous system (80), and activation of GLP-1 receptors in other organs or systems may result in unpredictable responses (81). Notably, the most common adverse reaction was digestive system adverse reactions (82). Most GI AEs are mild or moderate and can be resolved independently without intervention within a few weeks or months (83, 84). However, some patients may not tolerate them. Some studies have shown that this variation may be due to genetic variation (85). Considering that the intolerable GI adverse reaction will occur relatively shortly after the injection, the present study did not restrict the dosing duration.

Researchers have been concerned about the gastrointestinal adverse effects caused by GLP-1RAs. The influence of these adverse reactions effects on efficacy is still controversial. Lean et al. concluded that patients with GI AEs have greater weight loss (86), while other studies pointed out that nausea caused by GLP-1RAs is not significantly correlated with weight loss (87). Due to the remarkable efficacy of GLP-1RAs, it is increasingly used in clinical practice. Since we cannot prove a positive effect of gastrointestinal adverse effects on efficacy, should we consider sparing patients such suffering?

This is the first Network Meta-Analysis based on the number of sample withdrawals due to intolerable GI AEs by GLP-1RAs. The present study found that GLP-1RAs had a higher risk of intolerance than Insulin, SGLT2i, DPP4i, TZD, and Placebo. Among the GLP-1RA drugs, Liraglutide or Semaglutide had a higher risk of intolerant GI AE, and Dulaglutide had the lowest chance. Our findings are consistent with Alatorre et al. (88), who found a lower rate of adverse discontinuation with Dulaglutide. In a real-world study (89) using the disproportionality analysis model, it was also shown that Semaglutide and Liraglutide had the highest rate of adverse reactions among several GLP-1RAs. Still, in this literature, the authors also concluded that the incidence of GI adverse reactions with Dulaglutide was also higher. This differs from our findings, and we consider that this may be related to the fact that the studies we included were RCTs and not real-world studies and that the investigation by Li Chen et al. was based on the FDA adverse event reporting database, which differs from the data we included. Therefore, we believe that under both computational models, it can be assumed that Semaglutide and Liraglutide are more likely to cause intolerable GI adverse reactions in users. Dulaglutide causes GI adverse reactions but has a lower probability of being unbearable. In our study, we also found that there did not appear to be a statistically significant difference in intolerable GI AEs caused by Semaglutide weekly versus oral formulations. At the same time, the incidence of intolerable GI AEs caused by Semaglutide was positively correlated with the dose. In short, the higher the dose, the more the side effects.

Semaglutide, developed based on Liraglutide (90), showed similar results in this study. Semaglutide shares 94% identity with human GLP-1 (91), while Exenatide showed 53% homology to human-derived GLP-1 (92). However, Dulaglutide is also a human-derived GLP-1RA agent, indicating that Dulaglutide shares more than 90% identity with human GLP-1, so the intolerable GI AEs do not appear to be associated with an autoimmune response. Meanwhile, the GI AEs responsible for the intolerability of Semaglutide were unrelated to weekly or oral formulations. Drucker et al. (93) found that compared with the daily formula, the weekly recipe of exenatide has a lower risk of gastrointestinal adverse events, which is consistent with our research results. They also found no association between two different dosage forms of Exenatide and the production of corresponding anti-exenatide antibodies. So what accounts for the difference in intolerable GI AEs between Semaglutide, Liraglutide, and Dulaglutide? We hypothesized that the incidence of intolerable GI adverse effects caused by GLP-1RA might be related to the degree of central appetite suppression because endogenous GLP-1 has a very short half-life in the body (94). It mainly acts in a paracrine form, while exogenous GLP-1 can cross the blood-brain barrier (95), bind to GLP-1 receptors in the brain, and stimulate continuously, unlike endogenous GLP-1. The sensation of satiety is caused by the stimulation of GLP-1 receptors in the brain by GLP-1 RAs entering the blood-brain barrier. Currently, the brain-gut axis is thought to be associated with several functional gastrointestinal disorders (96). The physiological responses produced by the brain in response to GLP-1RA stimulation affect the gastrointestinal tract *via* the brain-gut axis. The gastrointestinal tract may attenuate this feeling of satiety through specific reactions. We hypothesized that the stronger the appetite suppression of GLP-1RA preparations, the more likely it is to lead to intolerable gastrointestinal adverse effects. However, this is only our conjecture and needs to be verified by further experiments.

Several methods were suggested to reduce the risk of GI AEs caused by GLP-1RAs. Choosing a small dose for the initial injection may reduce the risk of GI AEs (97), so pharmaceutical companies have developed a combination of GLP-1RA and insulin as a way to reduce the amount of GLP-1RA used (98, 99). This can both reduce the risk of hypoglycemic response caused by insulin and allow for better efficacy of GLP-1RAs. There are also different methods of injection to use Exenatide to reduce the incidence of intolerable GI AEs. However, this difference was not observed in our study.

Our study has some shortcomings. First of all, almost all the studies were lack of the dose of GLP-1RA when it was discontinued. Therefore, the current study could not conduct further Network Meta-Analysis according to the dose or the course of medication. Secondly, more than 25% of the RCTs included in this study had a sample size of fewer than 100 people, which made the OR and 95%CI of some outcomes with low accuracy; Thirdly, this study did not combine the curative effect with the comprehensive analysis of GLP-1RAs, but only analyzed from the direction of adverse reactions of the digestive tract; Fourthly, due to the lack of data, we only analyzed the correlation between the adverse reactions caused by Semaglutide and the dose. Whether other GLP-1RAs are consistent with semaglutide requires further validation in subsequent studies.

In conclusion, no single GLP-1RA has been proven to be superior across the board to the others. Each of these GLP-1RAs has its own merits (100), and physicians have the flexibility to choose the appropriate medicine based on the patient’s actual situation. In terms of our findings, we recommend Dulaglutide, which has a low risk of intolerable GI AEs.

## Conclusion

In conclusion, we found that Dulaglutide had the lowest risk of causing intolerable GI AEs. In contrast, Semaglutide and Liraglutide were associated with a higher risk of these AEs. We ruled out the possibility that the difference was due to drug homology or dosage form. However, no single GLP-1RA has been shown to be superior to the others. Each GLP-1RA has its advantages, and physicians have the flexibility to choose the appropriate drug according to the actual situation of the patient. In the future, we hope that more researchers will pay attention to these patients with adverse reactions in clinical trials, record their detailed data, and verify the reliability of our findings.

## Data availability statement

The original contributions presented in the study are included in the article/**Supplementary Material**. Further inquiries can be directed to the corresponding author.

## Author contributions

ZZ designed the network meta-analysis. ZZ, QZ, YC, SL, and YT selected the eligible articles. ZZ and XZ abstracted the data. ZZ and JY analyzed the data. ZZ wrote the paper. ZZ, QZ, YT, YC, XZ, SL, JY interpreted the results and all authors approved submitting the final manuscript.
