# Corrigendum: GLP-1RAs caused gastrointestinal adverse reactions of drug withdrawal: a system review and network meta-analysis

**DOI:** 10.3389/fendo.2023.1270115

**Published:** 2023-10-09

**Authors:** Ziqi Zhang, Qiling Zhang, Ying Tan, Yu Chen, Xiqiao Zhou, Su Liu, Jiangyi Yu

**Affiliations:** ^1^ Department of Endocrinology, Jiangsu Provincial Hospital of Chinese Medicine, Affiliated Hospital of Nanjing University of Traditional Chinese Medicine, Nanjing, Jiangsu, China; ^2^ The First Clinical Medical College of Nanjing University of Chinese Medicine, Nanjing, China

**Keywords:** glucagon-like peptide-1 receptor agonist, intolerance, gastrointestinal adverse effects, network meta-analysis, Dulaglutide, Liraglutide, Semaglutide

**Incorrect Affiliation**

In the published article, there was an error in affiliation 1. Instead of “The First Clinical Medical College of Nanjing University of Chinese Medicine, Nanjing, China”, it should be “Department of Endocrinology, Jiangsu Provincial Hospital of Chinese Medicine, Affiliated Hospital of Nanjing University of Traditional Chinese Medicine, Nanjing, Jiangsu, China”.

In the published article, there was an error in affiliation 2. Instead of “Department of Endocrinology, Jiangsu Provincial Hospital of Traditional Chinese Medicine, Affiliated Hospital of Nanjing University of Traditional Chinese Medicine, Nanjing, Jiangsu Province, China”, it should be “The First Clinical Medical College of Nanjing University of Chinese Medicine, Nanjing, China”.

The authors apologize for this error and state that this does not change the scientific conclusions of the article in any way. The original article has been updated.

**Additional Affiliation(s)**

In the published article, there was an error regarding the affiliation(s) for Ziqi Zhang, Qiling Zhang, qi Zhang, Qiling Zhang, Ying Tan, Yu Chen. As well as having affiliation 2, they should also have *1 Department of Endocrinology, Jiangsu Provincial Hospital of Chinese Medicine, Affiliated Hospital of Nanjing University of Traditional Chinese Medicine, Nanjing, Jiangsu, China*.

The authors apologize for this error and state that this does not change the scientific conclusions of the article in any way. The original article has been updated.

