# Supplementary material for: GLP-1RAs caused gastrointestinal adverse reactions of drug withdrawal: a system review and network meta-analysis
Source: Front Endocrinol (Lausanne). 2023 Jul 6;14:1149328. doi: 10.3389/fendo.2023.1149328 (PMC10359616; doi:10.3389/fendo.2023.1149328)
Supplement: Supplementary file 1 [file DataSheet_1.pdf]

STEP1

loop inconsistency test

Loop	IF	seIF	z_value	p_value	CI_95	Loop_Heterog_tau2
GLP1RA-Met-TZD	1.850	2.259	0.819	0.413	(0.00,6.28)	0.000
Met-DPP4-TZD	1.615	3.230	0.500	0.617	(0.00,7.95)	0.000
GLP1RA-Met-DPP4	1.566	2.125	0.737	0.461	(0.00,5.73)	0.041
GLP1RA-DPP4-TZD	1.273	1.588	0.801	0.423	(0.00,4.39)	0.000
GLP1RA-SU-DPP4	0.562	2.224	0.253	0.800	(0.00,4.92)	0.229
GLP1RA-Insulin-TZD	0.196	2.230	0.088	0.930	(0.00,4.57)	0.000
GLP1RA-Placebo-Blank	0.142	2.125	0.067	0.947	(0.00,4.31)	0.028

Loop	ROR	z_value	p_value	CI_95	Loop_Heterog_tau2
A-B-G	6.357	0.819	0.413	(1.00,532.42)	0.000
B-F-G	5.030	0.500	0.617	(1.00,2824.40)	0.000
A-B-F	4.789	0.737	0.461	(1.00,308.47)	0.041
A-F-G	3.572	0.801	0.423	(1.00,80.34)	0.000
A-D-F	1.755	0.253	0.800	(1.00,137.26)	0.229
A-C-G	1.217	0.088	0.930	(1.00,96.34)	0.000
A-H-I	1.153	0.067	0.947	(1.00,74.18)	0.028

node-splitting method

Side	Direct		Indirect		Difference		
	Coef.	Std. Err.	Coef.	Std. Err.	Coef.	Std. Err.	P>|z|
A B *	-.4237474	.5610843	-3.255045	2.776536	2.831298	2.78038	0.309
A C *	-2.025443	.4087778	.1941967	3.761243	-2.21964	3.747276	0.554
A D *	-9.11e-12	1.264979	-1.148454	3.322291	1.148454	3.554967	0.747
A E	.	.	.	.	.	.	.
A F *	-1.439496	.4490288	-1.119866	2.628157	-.3196301	2.641342	0.904
A G *	-1.747559	.9147766	-3.170195	1.622234	1.422636	1.553644	0.360
A H *	-1.574695	.1733766	-3.331862	3.540364	1.757167	3.539859	0.620
A I *	-1.452411	.6128601	-3.236787	3.316673	1.784376	3.319423	0.591
B F	.4107316	2.037319	-1.134499	.7531303	1.54523	2.172089	0.477
B G	.4103789	2.036915	-2.088349	1.198196	2.498728	2.363109	0.290
C G	.0145775	2.039847	.1128567	1.115522	-.0982791	2.324937	0.966
D F *	-.8979416	1.716675	-2.046395	2.645225	1.148454	3.554967	0.747
F G	-1.413395	1.184618	.692614	1.3176	-2.106009	1.633152	0.197
H I	2.86e-13	2.065791	.101734	.6632065	-.101734	2.169639	0.963

* Warning: all the evidence about these contrasts comes from the trials which directly compare them.

Inconsistency Model

chi2(9) = 6.09
Prob > chi2 = 0.7310

SUCRA

Treatm~t	SUCRA	PrBest	MeanRank
GLP1RA	91.8	44.1	1.7
Met	78.0	12.6	2.8
Insulin	22.6	0.0	7.2
SU	79.3	42.2	2.7
SGLT2	13.4	0.0	7.9
DPP4	48.0	0.0	5.2
TZD	29.8	0.9	6.6
Placebo	41.9	0.0	5.6
Blank	45.3	0.3	5.4

study and Rank	A	B	C	D	E	F	G	H	I
13									
Best	44.1	12.6	0.0	42.2	0.0	0.0	0.9	0.0	0.3
2nd	46.5	29.4	0.1	16.7	0.3	1.6	2.2	0.1	3.1
3rd	9.3	38.9	0.9	18.1	1.2	10.1	6.6	2.4	12.5
4th	0.2	11.9	3.9	7.2	3.4	26.5	11.9	12.8	22.3
5th	0.0	4.3	7.0	4.1	3.9	23.7	9.8	30.4	16.9
6th	0.0	1.7	13.9	3.3	6.4	18.0	9.6	31.9	15.2
7th	0.0	0.9	26.5	3.3	11.7	12.5	14.1	17.2	13.8
8th	0.0	0.2	32.7	2.7	21.9	6.0	21.0	4.7	10.8
Worst	0.0	0.1	15.1	2.3	51.1	1.6	24.0	0.5	5.1

STEP2

loop inconsistency test

Loop	IF	seIF	z_value	p_value	CI_95	Loop_Heterog_tau2
Liraglutide-Dulaglutide-DPP4i	3.912	2.229	1.755	0.079	(0.00,8.28)	0.000

Exenatide-Dulaglutide-DPP4i-Placebo	2.923	1.720	1.699	0.089	(0.00,6.29)	0.000
Dulaglutide-Met-Insulin-TZD	2.726	3.063	0.890	0.373	(0.00,8.73)	0.000
Dulaglutide-Semaglutide-Insulin-DPP4i	1.817	2.290	0.793	0.428	(0.00,6.31)	0.000
Exenatide-Met-TZD	1.801	2.397	0.751	0.452	(0.00,6.50)	0.000
Dulaglutide-Insulin-DPP4i-TZD	1.701	2.894	0.588	0.557	(0.00,7.37)	0.000
Exenatide-Dulaglutide-Insulin-DPP4i	1.685	1.898	0.888	0.375	(0.00,5.41)	0.000
Exenatide-Semaglutide-Insulin-Placebo	1.662	1.556	1.068	0.285	(0.00,4.71)	0.000
Met-DPP4i-TZD	1.615	3.230	0.500	0.617	(0.00,7.95)	0.000
Exenatide-Dulaglutide-Met-Insulin	1.609	1.512	1.064	0.287	(0.00,4.57)	0.000
Exenatide-Semaglutide-DPP4i-Placebo	1.530	0.911	1.680	0.093	(0.00,3.32)	0.000
Liraglutide-Semaglutide-DPP4i-Placebo	1.458	1.663	0.877	0.381	(0.00,4.72)	0.104
Liraglutide-Dulaglutide-Insulin	1.425	1.479	0.964	0.335	(0.00,4.32)	0.000
Dulaglutide-Semaglutide-DPP4i-Placebo	1.392	1.701	0.819	0.413	(0.00,4.73)	0.000
Exenatide-Dulaglutide-Insulin-Placebo	1.238	1.245	0.994	0.320	(0.00,3.68)	0.000
Exenatide-Liraglutide-Placebo	1.020	0.542	1.883	0.060	(0.00,2.08)	0.050
Exenatide-Liraglutide-DPP4i	0.991	1.604	0.618	0.536	(0.00,4.14)	0.000
Liraglutide-Met-DPP4i	0.921	2.934	0.314	0.754	(0.00,6.67)	0.000
Liraglutide-Semaglutide-Insulin-Placebo	0.885	1.672	0.529	0.597	(0.00,4.16)	0.044
Liraglutide-Met-Insulin-TZD	0.874	3.340	0.262	0.794	(0.00,7.42)	0.000
Liraglutide-Dulaglutide-Placebo	0.873	1.170	0.746	0.456	(0.00,3.17)	0.005
Liraglutide-Insulin-DPP4i-TZD	0.785	2.846	0.276	0.783	(0.00,6.36)	0.000
Liraglutide-Semaglutide-Insulin-DPP4i	0.670	2.230	0.300	0.764	(0.00,5.04)	0.000
Exenatide-DPP4i-TZD	0.549	1.899	0.289	0.772	(0.00,4.27)	0.000
Exenatide-SU-DPP4i	0.457	2.180	0.210	0.834	(0.00,4.73)	[illegible]

						0.000
Exenatide-Insulin-TZD	0.455	2.280	0.200	0.842	(0.00,4.92)	0.000
Liraglutide-Dulaglutide-Met	0.428	2.006	0.213	0.831	(0.00,4.36)	0.000
Dulaglutide-Semaglutide-Insulin-Placebo	0.424	1.806	0.235	0.814	(0.00,3.96)	0.000
Exenatide-Dulaglutide-Met-Placebo	0.371	1.281	0.290	0.772	(0.00,2.88)	0.000
Exenatide-Liraglutide-Insulin	0.190	1.008	0.188	0.851	(0.00,2.16)	0.000
Exenatide-Semaglutide-Insulin-DPP4i	0.132	1.772	0.074	0.941	(0.00,3.61)	0.000
Semaglutide-Insulin-DPP4i-TZD	0.116	2.813	0.041	0.967	(0.00,5.63)	0.000
Exenatide-Placebo-Blank	0.111	2.121	0.052	0.958	(0.00,4.27)	0.005
Exenatide-Met-DPP4i	0.092	2.261	0.041	0.968	(0.00,4.52)	0.000
Exenatide-Liraglutide-Met	0.054	1.817	0.030	0.976	(0.00,3.62)	0.000

Loop	ROR	z_value	p_value	CI_95	Loop_Heterog_tau2
B-C-K	49.984	1.755	0.079	(1.00,3942.84)	0.000
C-G-K	30.533	1.322	0.186	(1.00,4846.28)	0.000
A-C-K-M	18.590	1.699	0.089	(1.00,540.90)	0.000
C-G-H-L	15.278	0.890	0.373	(1.00,6185.99)	0.000
C-D-H-K	6.153	0.793	0.428	(1.00,547.70)	0.000
A-G-L	6.058	0.751	0.452	(1.00,664.92)	0.000
C-H-K-L	5.481	0.588	0.557	(1.00,1593.04)	0.000
A-C-H-K	5.393	0.888	0.375	(1.00,222.69)	0.000
A-D-H-M	5.270	1.068	0.285	(1.00,111.14)	0.000
G-K-L	5.030	0.500	0.617	(1.00,2824.34)	0.000
A-C-G-H	4.998	1.064	0.287	(1.00,96.81)	0.000
A-D-K-M	4.619	1.680	0.093	(1.00,27.54)	0.000
B-D-K-M	4.298	0.877	0.381	(1.00,111.94)	0.104
B-C-H	4.157	0.964	0.335	(1.00,75.43)	0.000
C-D-K-M	4.025	0.819	0.413	(1.00,112.85)	0.000
A-C-H-M	3.447	0.994	0.320	(1.00,39.52)	0.000
A-B-M	2.774	1.883	0.060	(1.00,8.02)	0.050
A-B-K	2.695	0.618	0.536	(1.00,62.49)	0.000
B-G-K	2.511	0.314	0.754	(1.00,788.80)	0.000

B-D-H-M	2.422	0.529	0.597	(1.00,64.14)	0.044
B-G-H-L	2.396	0.262	0.794	(1.00,1670.04)	0.000
B-C-M	2.393	0.746	0.456	(1.00,23.70)	0.005
B-H-K-L	2.193	0.276	0.783	(1.00,580.66)	0.000
B-D-H-K	1.954	0.300	0.764	(1.00,154.50)	0.000
A-K-L	1.732	0.289	0.772	(1.00,71.59)	0.000
A-I-K	1.580	0.210	0.834	(1.00,113.28)	0.000
A-H-L	1.577	0.200	0.842	(1.00,137.66)	0.000
B-C-G	1.534	0.213	0.831	(1.00,78.15)	0.000
C-D-H-M	1.529	0.235	0.814	(1.00,52.62)	0.000
A-C-G-M	1.450	0.290	0.772	(1.00,17.84)	0.000
A-B-H	1.209	0.188	0.851	(1.00,8.71)	0.000
A-D-H-K	1.141	0.074	0.941	(1.00,36.80)	0.000
D-H-K-L	1.122	0.041	0.967	(1.00,278.30)	0.000
A-M-N	1.117	0.052	0.958	(1.00,71.38)	0.005
A-G-K	1.096	0.041	0.968	(1.00,92.08)	0.000
A-B-G	1.055	0.030	0.976	(1.00,37.19)	0.000

+--+

node-splitting method

Side	Direct		Indirect		Difference		
	Coef.	Std. Err.	Coef.	Std. Err.	Coef.	Std. Err.	P>|z|
A B	1.183487	.423013	.3442286	.3773265	.8392586	.5633891	0.136
A F	.	.	.	.	.	.	.
A G	-.7040413	.8072199	-.5045074	.8179191	-.1995339	1.126351	0.859
A H	-1.604549	.5903014	-2.160989	.6040188	.5564398	.8351691	0.505
A I *	3.08e-10	1.244699	-.5960247	3.308097	.5960247	3.534513	0.866
A K *	-.5299589	.6644503	-1.717559	.6020594	1.1876	.8898376	0.182
A L *	-1.75214	.9069505	-2.788724	1.642966	1.036584	1.57048	0.509
A M	-1.472637	.2464666	-.4663911	.4484715	-1.006246	.5203232	0.053
A N *	-1.555421	.6041542	-1.49557	3.312354	-.0598501	3.316434	0.986
B C	-1.950359	.9704285	-.9763099	.5502653	-.9740494	1.108213	0.379
B G	-2.032153	1.634438	-1.185297	.6578561	-.846856	1.761852	0.631
B H	-2.491118	.7718886	-2.615243	.5491952	.1241251	.9463774	0.896
B K	-.6931404	1.451732	-2.03505	.5314818	1.34191	1.545957	0.385
B M	-1.66771	.3521489	-2.461433	.4804096	.7937222	.6070325	0.191
C G	.4148294	.828465	-.6615253	.8747227	1.076355	1.204776	0.372
C H	-1.912641	.904379	-1.066344	.6685111	-.8462963	1.124371	0.452
C K	-2.585267	1.477088	-.2909707	.6554469	-2.294297	1.615982	0.156
C M	-.52243	.6588761	-.9326053	.6594215	.4101753	.9572767	0.668
D H	-3.468832	1.458123	-2.3981	.5297036	-1.070732	1.551354	0.490
D J *	-2.466712	.6789759	-3.758701	199.9245	1.291989	199.9238	0.995
D K	-2.329909	.6574917	-1.337494	.6436675	-.992415	.9201084	0.281
D M	-1.769405	.2488008	-2.856996	.7697442	1.087591	.8098771	0.179
E M *	-1.722973	1.078311	-.7541222	200.019	-.9688509	200.0272	0.996
G K	.4188531	2.025338	-.7097333	.7586362	1.128586	2.162784	0.602

G L	.4184009	2.024964	-1.871448	1.203848	2.289849	2.355702	0.331
H L	.0145825	2.027049	-.0051992	1.111525	.0197816	2.311792	0.993
I K *	-.8979416	1.701786	-1.493966	2.627375	.5960244	3.534513	0.866
K L	-1.389407	1.174132	.2252102	1.330093	-1.614617	1.637627	0.324
M N	6.37e-10	2.053226	-.3497045	.6675124	.3497045	2.159007	0.871

* Warning: all the evidence about these contrasts comes from the trials which directly compare them.

Inconsistency Model

chi2(22) = 11.88
Prob > chi2 = 0.9598

SUCRA

Treatment	SUCRA	PrBest	MeanRank
Exenatide	66.3	0.0	5.4
Liraglutide	87.7	18.5	2.6
Dulaglutide	52.7	0.1	7.1
Semaglutide	85.8	14.8	2.8
Tirzepatide	76.4	31.5	4.1
Taspoglutide	85.4	18.5	2.9
Met	50.0	0.4	7.5
Insulin	12.4	0.0	12.4
SU	63.4	16.3	5.8
SGLT2i	16.7	0.0	11.8
DPP4i	32.8	0.0	9.7
TZD	17.2	0.1	11.8
Placebo	30.9	0.0	10.0
Blank	22.4	0.0	11.1

study and Rank	A	B	C	D	E	F	G	H	I	J	K	L	M	N
13														
Best	0.0	18.5	0.1	14.8	31.5	18.5	0.4	0.0	16.3	0.0	0.0	0.1	0.0	0.0
2nd	0.1	30.8	0.2	26.6	10.1	23.4	0.6	0.0	8.1	0.0	0.0	0.1	0.0	0.0
3rd	1.2	28.4	0.8	29.1	8.0	24.2	1.6	0.0	6.2	0.1	0.0	0.2	0.0	0.1
4th	15.0	16.9	2.7	20.5	10.5	21.5	3.6	0.0	8.5	0.1	0.1	0.5	0.0	0.1
5th	40.0	4.9	8.2	7.5	9.8	9.2	7.7	0.0	10.4	0.5	0.5	0.9	0.0	0.5
6th	33.0	0.4	19.9	1.2	9.0	2.3	15.7	0.0	10.8	1.4	2.2	2.1	0.2	1.7
7th	9.1	0.1	30.2	0.3	7.1	0.6	23.6	0.3	9.6	2.7	7.2	3.4	1.8	4.1
8th	1.6	0.0	22.8	0.1	5.0	0.2	20.1	1.0	9.0	4.5	14.6	5.2	9.0	7.1
[illegible]	[illegible]	[illegible]	[illegible]	[illegible]	[illegible]	[illegible]	[illegible]	[illegible]	[illegible]	[illegible]	[illegible]	[illegible]	[illegible]	[illegible]

9th	0.1	0.0	8.8	0.0	2.8	0.0	10.8	2.8	5.3	7.0	21.5	6.8	24.0	10.1
10th	0.0	0.0	3.7	0.0	2.0	0.0	7.0	5.4	3.8	7.7	20.8	7.1	31.2	11.2
11th	0.0	0.0	1.6	0.0	1.4	0.0	4.4	12.6	3.4	11.7	16.3	9.6	23.3	15.8
12th	0.0	0.0	0.8	0.0	1.5	0.0	2.7	24.0	3.4	15.9	10.7	12.7	8.8	19.5
13th	0.0	0.0	0.2	0.0	0.9	0.0	1.3	32.2	2.5	20.7	5.0	18.0	1.7	17.5
Worst	0.0	0.0	0.0	0.0	0.5	0.0	0.4	21.7	2.7	27.8	1.2	33.4	0.1	12.2

STEP3
loop inconsistency test

Loop	IF	seIF	z_value	p_value	CI_95	Loop_Heterog_tau2
Efpeglenatide qm-Placebo-Liraglutide	2.803	2.822	0.993	0.321	(0.00,8.33)	0.000
Exenatide bid-Met-TZD	2.417	2.633	0.918	0.359	(0.00,7.58)	0.000
Exenatide bid-Exenatide qw-TZD-Placebo	2.161	2.269	0.952	0.341	(0.00,6.61)	0.583
Exenatide qw-Placebo-Blank	2.116	2.505	0.845	0.398	(0.00,7.03)	0.000
Exenatide qw-Semaglutide qw-DPP4i-Placebo	2.080	1.331	1.563	0.118	(0.00,4.69)	0.000
Exenatide bid-Lixisenatide-Insulin-Placebo	1.921	1.938	0.991	0.322	(0.00,5.72)	0.000
Exenatide qw-Placebo-Liraglutide	1.919	1.320	1.454	0.146	(0.00,4.51)	0.000
Exenatide bid-Exenatide qw-Insulin-Placebo	1.719	1.444	1.190	0.234	(0.00,4.55)	0.312
Exenatide bid-Exenatide qw-Met-Blank	1.547	2.885	0.536	0.592	(0.00,7.20)	0.000
Efpeglenatide qw-Efpeglenatide qm-Liraglutide	1.480	2.466	0.600	0.548	(0.00,6.31)	0.000
Lixisenatide-Semaglutide qw-DPP4i-Placebo	1.463	1.239	1.181	0.238	(0.00,3.89)	0.000
Exenatide bid-Exenatide qw-Met-Insulin	1.391	2.152	0.646	0.518	(0.00,5.61)	0.000
Efpeglenatide qw-Efpeglenatide qm-Placebo	1.323	2.811	0.471	0.638	(0.00,6.83)	0.000
Exenatide bid-Exenatide qw-DPP4i-Placebo	1.322	2.272	0.582	0.560	(0.00,5.77)	0.559
Lixisenatide-Placebo-Blank	1.188	2.638	0.451	0.652	(0.00,6.36)	0.000

						[illegible]
Exenatide bid-Insulin-TZD	1.130	2.647	0.427	0.669	(0.00,6.32)	0.000
Exenatide bid-Lixisenatide-DPP4i-Placebo	1.046	1.890	0.553	0.580	(0.00,4.75)	0.052
Exenatide qw-Met-TZD	0.907	3.016	0.301	0.764	(0.00,6.82)	0.000
Exenatide bid-DPP4i-TZD	0.809	2.541	0.318	0.750	(0.00,5.79)	0.000
Lixisenatide-Insulin-DPP4i-TZD	0.806	2.844	0.283	0.777	(0.00,6.38)	0.000
Lixisenatide-Efpeglenatide qw-Placebo	0.804	2.074	0.388	0.698	(0.00,4.87)	0.000
Exenatide bid-Lixisenatide-Insulin-DPP4i	0.803	2.444	0.328	0.743	(0.00,5.59)	0.000
Exenatide bid-Lixisenatide-Insulin-Blank	0.709	2.888	0.245	0.806	(0.00,6.37)	0.000
Exenatide qw-Met-DPP4i	0.708	2.738	0.259	0.796	(0.00,6.07)	0.000
Exenatide qw-Lixisenatide-DPP4i-Placebo	0.618	1.072	0.576	0.564	(0.00,2.72)	0.000
Exenatide bid-Met-DPP4i	0.600	2.754	0.218	0.828	(0.00,6.00)	0.000
Exenatide qw-Lixisenatide-Insulin-DPP4i	0.554	1.941	0.286	0.775	(0.00,4.36)	0.000
Exenatide bid-Semaglutide qw-DPP4i-Placebo	0.530	2.606	0.203	0.839	(0.00,5.64)	1.002
Efpeglenatide qw-Placebo-Liraglutide	.	.	.	.		0.000
Exenatide bid-Placebo-Blank	0.350	3.243	0.108	0.914	(0.00,6.71)	1.540
Exenatide qw-Insulin-TZD	0.263	2.421	0.109	0.913	(0.00,5.01)	0.000
Exenatide bid-Exenatide qw-Insulin-DPP4i	0.248	2.146	0.116	0.908	(0.00,4.46)	0.000
Exenatide bid-Exenatide qw-Insulin-Blank	0.156	2.461	0.063	0.949	(0.00,4.98)	0.000
Exenatide bid-Exenatide qw-Met-Placebo	0.141	2.477	0.057	0.955	(0.00,5.00)	0.814
Exenatide qw-Lixisenatide-Insulin-Placebo	0.063	1.898	0.033	0.973	(0.00,3.78)	0.000
Exenatide qw-DPP4i-TZD	.	.	.	.		0.000
Exenatide bid-SU-DPP4i	.	.	.	.		0.000

+---+

*** Note: Loop Exenatide bid-SU-DPP4i is formed only by multi-arm trial(s) - Consistent by definition

*** Note: Loop Exenatide qw-DPP4i-TZD is formed only by multi-arm trial(s) - Consistent by definition

*** Note: Loop Efpeglenatide qw-Placebo-Liraglutide is formed only by multi-arm trial(s) - Consistent by definition

Loop	ROR	z_value	p_value	CI_95	Loop_Heterog_tau2
H-Q-S	16.493	0.993	0.321	(1.00,4164.39)	0.000
B-K-P	11.216	0.918	0.359	(1.00,1953.89)	0.000
B-C-P-Q	8.676	0.952	0.341	(1.00,741.35)	0.583
C-I-O-Q	8.008	1.563	0.118	(1.00,108.72)	0.000
B-F-L-Q	6.830	0.991	0.322	(1.00,304.98)	0.000
C-Q-S	6.812	1.454	0.146	(1.00,90.49)	0.000
B-C-L-Q	5.580	1.190	0.234	(1.00,94.64)	0.312
K-O-P	5.030	0.500	0.617	(1.00,2824.34)	0.000
B-C-K-R	4.698	0.536	0.592	(1.00,1341.64)	0.000
G-H-S	4.393	0.600	0.548	(1.00,552.04)	0.000
F-I-O-Q	4.317	1.181	0.238	(1.00,48.94)	0.000
B-C-K-L	4.020	0.646	0.518	(1.00,272.90)	0.000
G-H-Q	3.754	0.471	0.638	(1.00,926.62)	0.000
B-C-O-Q	3.753	0.582	0.560	(1.00,322.07)	0.559
F-Q-R	3.282	0.451	0.652	(1.00,576.96)	0.000
B-L-P	3.096	0.427	0.669	(1.00,554.28)	0.000
B-F-O-Q	2.845	0.553	0.580	(1.00,115.70)	0.052
C-K-P	2.478	0.301	0.764	(1.00,914.98)	0.000
B-O-P	2.245	0.318	0.750	(1.00,326.51)	0.000
F-L-O-P	2.239	0.283	0.777	(1.00,589.84)	0.000
F-G-Q	2.234	0.388	0.698	(1.00,130.17)	0.000
B-F-L-O	2.231	0.328	0.743	(1.00,268.47)	0.000
B-F-L-R	2.032	0.245	0.806	(1.00,584.07)	0.000
C-K-O	2.030	0.259	0.796	(1.00,434.64)	0.000
C-F-O-Q	1.855	0.576	0.564	(1.00,15.16)	0.000
B-K-O	1.822	0.218	0.828	(1.00,402.55)	0.000
C-F-L-O	1.741	0.286	0.775	(1.00,78.18)	0.000
B-I-O-Q	1.699	0.203	0.839	(1.00,280.73)	1.002
G-Q-S	.	.	.		0.000
B-Q-R	1.419	0.108	0.914	(1.00,816.75)	1.540
C-L-P	1.301	0.109	0.913	(1.00,149.62)	0.000
B-C-L-O	1.282	0.116	0.908	(1.00,86.06)	0.000
B-C-L-R	1.169	0.063	0.949	(1.00,145.48)	0.000
B-C-K-Q	1.152	0.057	0.955	(1.00,147.95)	0.814
C-F-L-Q	1.065	[illegible]	[illegible]	[illegible]	[illegible]

C-F-L-Q	1.065	0.033	0.973	(1.00,44.00)	0.000
C-O-P	.	.	.		0.000
B-M-O	.	.	.		0.000
+---+

*** Note: Loop B-M-O is formed only by multi-arm trial(s) - Consistent by definition

*** Note: Loop C-O-P is formed only by multi-arm trial(s) - Consistent by definition

*** Note: Loop G-Q-S is formed only by multi-arm trial(s) - Consistent by definition

node-splitting method

<table>
<tr><th>Side</th><th>Direct</th><th></th><th>Indirect</th><th></th><th>Difference</th><th></th><th></th></tr>
<tr><th></th><th>Coef.</th><th>Std. Err.</th><th>Coef.</th><th>Std. Err.</th><th>Coef.</th><th>Std. Err.</th><th>P>|z|</th></tr>
<tr><td>A B *</td><td>-.6340232</td><td>.5357435</td><td>-.3114992</td><td>16.0155</td><td>-.322524</td><td>16.02446</td><td>0.984</td></tr>
<tr><td>B K</td><td>-.7167163</td><td>1.023365</td><td>-1.449214</td><td>1.706989</td><td>.7324975</td><td>1.99022</td><td>0.713</td></tr>
<tr><td>B L</td><td>-2.001274</td><td>.8058184</td><td>-.5124432</td><td>.9621489</td><td>-1.488831</td><td>1.244817</td><td>0.232</td></tr>
<tr><td>B M *</td><td>-.0000261</td><td>1.308975</td><td>.5495031</td><td>3.514504</td><td>-.5495292</td><td>3.750237</td><td>0.884</td></tr>
<tr><td>B O *</td><td>-.8980111</td><td>1.749308</td><td>-.6232881</td><td>.675976</td><td>-.274723</td><td>1.875355</td><td>0.884</td></tr>
<tr><td>B P</td><td>-2.957835</td><td>1.44999</td><td>-1.017523</td><td>1.183823</td><td>-1.940312</td><td>1.787782</td><td>0.278</td></tr>
<tr><td>B Q</td><td>-1.241566</td><td>.493787</td><td>-2.482947</td><td>.8703459</td><td>1.241381</td><td>1.03252</td><td>0.229</td></tr>
<tr><td>B R</td><td>-.9985178</td><td>1.708203</td><td>-.4638434</td><td>1.194822</td><td>-.5346744</td><td>2.026643</td><td>0.792</td></tr>
<tr><td>C K</td><td>-.8866376</td><td>1.662571</td><td>-.7654319</td><td>1.138681</td><td>-.1212057</td><td>1.945252</td><td>0.950</td></tr>
<tr><td>C L</td><td>-.9435854</td><td>.8482679</td><td>-1.73223</td><td>.9444197</td><td>.7886441</td><td>1.276378</td><td>0.537</td></tr>
<tr><td>C O</td><td>-.1325979</td><td>.8761458</td><td>-.8490549</td><td>.7300753</td><td>.716457</td><td>1.139731</td><td>0.530</td></tr>
<tr><td>C P</td><td>-1.178432</td><td>1.133877</td><td>-2.501222</td><td>1.393776</td><td>1.32279</td><td>1.639103</td><td>0.420</td></tr>
<tr><td>C Q</td><td>-2.098118</td><td>.3555121</td><td>-.59456</td><td>.6043105</td><td>-1.503558</td><td>.7044692</td><td>0.033</td></tr>
<tr><td>C R</td><td>-.0001178</td><td>1.527214</td><td>-.8986207</td><td>1.267742</td><td>.8985029</td><td>1.984801</td><td>0.651</td></tr>
<tr><td>C S</td><td>1.155241</td><td>.6586116</td><td>.0405092</td><td>1.171315</td><td>1.114732</td><td>1.343778</td><td>0.407</td></tr>
<tr><td>D Q *</td><td>-.5378543</td><td>1.660396</td><td>-3.851998</td><td>200.0159</td><td>3.314143</td><td>200.0353</td><td>0.987</td></tr>
<tr><td>E Q *</td><td>-3.157113</td><td>1.516988</td><td>-1.232481</td><td>200.0218</td><td>-1.924632</td><td>200.0378</td><td>0.992</td></tr>
<tr><td>F G</td><td>-1.609399</td><td>1.642794</td><td>-1.701513</td><td>1.037516</td><td>.0921136</td><td>1.94297</td><td>0.962</td></tr>
<tr><td>F L</td><td>-1.67613</td><td>1.673941</td><td>-2.570489</td><td>.8174109</td><td>.8943586</td><td>1.862855</td><td>0.631</td></tr>
<tr><td>F O</td><td>-1.600195</td><td>.5159722</td><td>-1.861098</td><td>.8964719</td><td>.2609025</td><td>1.031381</td><td>0.800</td></tr>
<tr><td>F Q</td><td>-2.815818</td><td>.6918423</td><td>-2.285657</td><td>.7170963</td><td>-.5301616</td><td>.9964196</td><td>0.595</td></tr>
<tr><td>F R</td><td>-1.628028</td><td>1.635482</td><td>-1.632988</td><td>1.247891</td><td>.0049591</td><td>2.05716</td><td>0.998</td></tr>
<tr><td>G H *</td><td>1.175315</td><td>1.755344</td><td>1.326676</td><td>3.118581</td><td>-.1513602</td><td>3.931901</td><td>0.969</td></tr>
<tr><td>G Q *</td><td>-.3312742</td><td>1.182909</td><td>-1.64135</td><td>1.40702</td><td>1.310075</td><td>2.001282</td><td>0.513</td></tr>
<tr><td>G S *</td><td>1.276327</td><td>.7760029</td><td>2.311481</td><td>1.731799</td><td>-1.035154</td><td>1.892504</td><td>0.584</td></tr>
<tr><td>H Q *</td><td>-.7129498</td><td>1.756144</td><td>-5.510617</td><td>3.059264</td><td>4.797667</td><td>3.896806</td><td>0.218</td></tr>
<tr><td>H S *</td><td>-1.175573</td><td>1.745696</td><td>3.519158</td><td>2.959556</td><td>-4.694731</td><td>3.802794</td><td>0.217</td></tr>
<tr><td>I O</td><td>-2.329916</td><td>.761136</td><td>-1.111402</td><td>1.098145</td><td>-1.218514</td><td>1.336136</td><td>0.362</td></tr>
<tr><td>I Q</td><td>-2.259938</td><td>.919647</td><td>-3.478336</td><td>.9638049</td><td>1.218398</td><td>1.336178</td><td>0.362</td></tr>
<tr><td>J N *</td><td>-2.466712</td><td>.7862955</td><td>-1.033961</td><td>200.1146</td><td>-1.432752</td><td>200.1143</td><td>0.994</td></tr>
<tr><td>J Q *</td><td>-1.478493</td><td>.5464064</td><td>-2.33815</td><td>89.44131</td><td>.8596571</td><td>89.44309</td><td>0.992</td></tr>
<tr><td>K O</td><td>.4189452</td><td>2.067184</td><td>.1968963</td><td>1.159232</td><td>.2220489</td><td>2.370138</td><td>0.925</td></tr>
<tr><td>[illegible]</td><td>[illegible]</td><td>[illegible]</td><td>[illegible]</td><td>[illegible]</td><td>[illegible]</td><td>[illegible]</td><td>[illegible]</td></tr>
</table>

```
K P      .4183904   2.066924  -1.564209   1.522394   1.982599    2.56708  0.440
L P      .0145893   2.066841  -.5150149   1.235075   .5296042    2.40774  0.826
M O *   -.8979416   1.749364  -.3486747   2.883577  -.5492669   3.750853  0.884
O P     -1.209271    1.23319  -.9810498    1.45978   -.228221   1.801052  0.899
Q R     -8.20e-10   2.091192   1.187961   1.100193  -1.187961   2.362945  0.615
Q S *    1.409557   1.212485    2.77129   .7926605  -1.361732   1.465503  0.353
```

* Warning: all the evidence about these contrasts comes from the trials which directly compare them.

Inconsistency Model

```
          chi2( 17) =    5.86
        Prob > chi2 =    0.9941
```

SUCRA

```
  +---------------------------------------------+
  |         Treatment | SUCRA | PrBest | MeanRank |
  |-------------------+-------+--------+----------|
  |       Taspoglutide |  74.1 |    4.4 |      5.7 |
  |      Exenatide bid |  57.3 |    0.0 |      8.7 |
  |       Exenatide qw |  54.9 |    0.0 |      9.1 |
  |             PEX168 |  35.8 |    3.3 |     12.6 |
  |            ITCA650 |  83.8 |   43.5 |      3.9 |
  |       Lixisenatide |  83.2 |    5.5 |      4.0 |
  |   Efpeglenatide qw |  39.4 |    0.0 |     11.9 |
  |   Efpeglenatide qm |  68.4 |   13.6 |      6.7 |
  |      Semaglutide qw |  87.1 |   17.4 |      3.3 |
  | oral Semaglutide  |  55.3 |    0.2 |      9.0 |
  |                Met |  33.8 |    0.1 |     12.9 |
  |            Insulin |  20.8 |    0.0 |     15.3 |
  |                 SU |  58.0 |    6.3 |      8.6 |
  |             SGLT2i |   6.9 |    0.0 |     17.8 |
  |              DPP4i |  38.8 |    0.0 |     12.0 |
  |                TZD |  16.5 |    0.0 |     16.0 |
  |            Placebo |  16.5 |    0.0 |     16.0 |
  |              Blank |  41.3 |    0.5 |     11.6 |
  |        Liraglutide |  78.1 |    5.2 |      4.9 |
  +---------------------------------------------+
```

```
----------------------------------------------------------------------------------------------------------------------
study and |                                     Treatment
Rank      |   A    B    C    D    E    F    G    H    I    J    K    L    M    N    O    P    Q    R    S
----------+-----------------------------------------------------------------------------------------------------------
```

15 |
Best | 4.4 0.0 0.0 3.3 43.5 5.5 0.0 13.6 17.4 0.2 0.1 0.0 6.3 0.0 0.0 0.0 0.0 0.5 5.2
2nd | 9.0 0.1 0.1 3.7 13.1 14.9 0.1 11.9 26.0 1.0 0.2 0.0 7.2 0.0 0.0 0.1 0.0 1.1 11.7
3rd | 11.3 0.5 0.2 2.9 7.5 22.3 0.5 8.5 19.7 2.0 0.6 0.0 6.7 0.0 0.0 0.0 0.0 1.7 15.6
4th | 13.6 1.6 1.2 2.7 6.1 22.4 1.3 7.9 13.6 3.8 1.1 0.0 6.5 0.0 0.1 0.1 0.0 2.2 15.7
5th | 14.3 4.5 3.5 3.4 5.4 15.7 2.1 7.7 9.0 6.6 1.6 0.0 6.5 0.1 0.5 0.3 0.0 3.4 15.3
6th | 12.7 9.4 7.8 3.6 4.3 9.8 3.8 6.4 6.0 8.8 2.6 0.1 6.8 0.1 1.1 0.5 0.0 4.4 11.9
7th | 10.5 14.7 11.9 3.1 3.0 5.3 4.6 6.1 3.6 10.3 3.4 0.2 6.3 0.1 2.0 0.6 0.0 5.4 9.0
8th | 8.1 17.0 15.3 3.3 2.6 2.3 5.8 4.9 2.3 11.1 4.0 0.3 6.1 0.2 3.9 1.0 0.0 5.7 6.1
9th | 5.3 17.0 16.1 3.7 2.4 1.1 6.3 4.8 1.2 12.0 5.2 0.8 5.5 0.3 6.9 1.3 0.0 6.0 4.2
10th | 4.2 14.3 16.4 3.9 1.9 0.5 8.2 4.6 0.7 11.0 5.3 1.5 5.5 0.4 9.8 1.7 0.0 7.5 2.5
11th | 2.8 10.1 13.1 4.2 2.2 0.2 9.6 3.8 0.4 10.1 8.0 3.2 5.7 0.6 13.6 2.8 0.2 7.8 1.5
12th | 1.6 6.2 8.2 4.5 1.6 0.0 11.0 4.4 0.1 8.8 8.9 5.6 5.7 1.2 17.7 3.7 0.9 9.1 0.8
13th | 1.2 2.9 4.1 5.0 2.0 0.0 11.4 4.0 0.1 6.3 10.1 9.1 5.4 2.0 18.1 5.2 3.1 9.5 0.5
14th | 0.6 1.2 1.7 5.8 1.3 0.0 11.0 3.1 0.0 4.6 11.6 13.1 5.0 3.0 12.8 7.1 9.3 8.8 0.1
15th | 0.3 0.3 0.4 6.5 0.8 0.0 8.6 2.4 0.0 2.3 9.9 15.3 4.2 3.6 8.1 9.4 19.7 8.2 0.0
16th | 0.1 0.1 0.1 5.9 0.7 0.0 6.2 1.9 0.0 0.9 9.4 17.3 3.2 5.6 3.6 11.2 27.4 6.4 0.0
17th | 0.0 0.0 0.0 7.3 0.7 0.0 5.0 1.7 0.0 0.3 8.4 16.9 2.7 9.5 1.3 14.6 25.8 5.6 0.0
18th | 0.0 0.0 0.0 11.5 0.6 0.0 3.3 1.5 0.0 0.0 6.6 12.1 3.4 21.7 0.5 22.2 12.1 4.5 0.0
Worst | 0.0 0.0 0.0 15.7 0.3 0.0 1.1 0.7 0.0 0.0 2.9 4.3 1.4 51.8 0.1 18.2 1.5 2.1 0.0

STEP4
loop inconsistency test

+--+

```
|                                     Loop |    IF |  seIF | z_value | p_value |       CI_95 | Loop_Heterog_tau2 |
|-----------------------------------------+-------+-------+---------+---------+-------------+-------------------|
|   Semaglutide 0.5mg-Semaglutide 1mg-DPP4i | 0.379 | 0.880 |   0.431 |   0.667 | (0.00,2.10) |             0.000 |
| Semaglutide 0.5mg-Semaglutide 1mg-Placebo | 0.247 | 0.449 |   0.549 |   0.583 | (0.00,1.13) |             0.000 |
| Semaglutide 0.5mg-Semaglutide 1mg-Insulin | 0.152 | 2.043 |   0.075 |   0.941 | (0.00,4.16) |             0.000 |

+---------------------------------------------------------------------------------------------------------------+
```

```
+---------------------------------------------------------------------+
|  Loop |   ROR | z_value | p_value |        CI_95 | Loop_Heterog_tau2 |
|-------+-------+---------+---------+--------------+-------------------|
| A-B-E | 1.461 |   0.431 |   0.667 |  (1.00,8.19) |             0.000 |
| A-B-D | 1.280 |   0.549 |   0.583 |  (1.00,3.09) |             0.000 |
| A-B-C | 1.165 |   0.075 |   0.941 | (1.00,63.82) |             0.000 |
+---------------------------------------------------------------------+
```

node-splitting method

Side	Direct		Indirect		Difference		
	Coef.	Std. Err.	Coef.	Std. Err.	Coef.	Std. Err.	P>|z|
A B	.	.	.	.	.	.	.
A C *	-3.160776	1.458835	-3.555979	1.618329	.395203	.9598153	0.681
A D *	-1.82034	.2826938	-2.290948	.5737178	.4706073	.575731	0.414
A E *	-2.253219	.6127507	-1.491297	.790205	-.7619215	.6384198	0.233
B C *	-3.717706	1.446585	-3.322503	1.650988	-.395203	.9598153	0.681
B D *	-2.343583	.2693367	-1.872976	.5926826	-.4706075	.575731	0.414
B E *	-2.401896	.6088614	-3.163817	.7991732	.7619215	.6384198	0.233

Inconsistency Model

chi2(2) =	0.82
Prob > chi2 =	0.6634

Treatment estimate (sm = 'OR', comparison: other treatments vs 'D'):

	OR	95%-CI	z	p-value
A	0.1528	[0.0894; 0.2612]	-6.87	< 0.0001
B	0.0996	[0.0592; 0.1676]	-8.69	< 0.0001
C	3.9072	[0.2276; 67.0713]	0.94	0.3475
D	.	.	.	.
E	1.2539	[0.3511; 4.4785]	0.35	0.7276

Quantifying heterogeneity / inconsistency:

tau^2 = 0; tau = 0; I^2 = 0% [0.0%; 79.2%]

[illegible]

```
Design-specific decomposition of within-designs Q statistic

     Design    Q df p-value
 D vs A vs B 1.62  2  0.4459
```

SUCRA

```
  +------------------------------------------+
  |        Treatment | SUCRA | PrBest | MeanRank |
  |-------------------+-------+--------+----------|
  | Semaglutide 0.5mg |  74.8 |    0.8 |      2.0 |
  |   Semaglutide 1mg |  99.6 |   98.6 |      1.0 |
  |           Insulin |  10.9 |    0.7 |      4.6 |
  |           Placebo |  36.5 |    0.0 |      3.5 |
  |             DPP4i |  28.2 |    0.0 |      3.9 |
  +------------------------------------------+
```

```
----------------------------------------
study and |          Treatment
Rank      |   A    B    C    D    E
----------+-----------------------------
1         |
     Best |  0.8  98.6   0.7   0.0   0.0
      2nd | 97.8   1.4   0.8   0.0   0.0
      3rd |  1.4   0.0  13.7  53.6  31.3
      4th |  0.0   0.0  11.1  38.7  50.1
    Worst |  0.0   0.0  73.8   7.7  18.6
----------------------------------------
```
